# Extensive Admixture Among Karst‐Obligate Salamanders Reveals Evidence of Recent Divergence and Gene Exchange Through Aquifers

**DOI:** 10.1002/ece3.70785

**Published:** 2025-01-09

**Authors:** Chris C. Nice, Katherine L. Bell, Zachariah Gompert, Lauren K. Lucas, James R. Ott, Ruben U. Tovar, Paul Crump, Peter H. Diaz

**Affiliations:** ^1^ Department of Biology, Population and Conservation Biology Program Texas State University San Marcos Texas USA; ^2^ Department of Biology University of Nevada Reno Nevada USA; ^3^ Department of Biology Utah State University Logan Utah USA; ^4^ Department of Integrative Biology The University of Texas at Austin Austin Texas USA; ^5^ Nongame and Rare Species Program, Wildlife Division, Texas Parks and Wildlife Department Austin Texas USA; ^6^ United States Fish and Wildlife Service, Texas Fish and Wildlife Conservation Office San Marcos Texas USA

**Keywords:** *Eurycea*, gene flow, genomic differentiation, Plethodontidae, population genomics

## Abstract

Karst ecosystems often contain extraordinary biodiversity, but the complex underground aquifers of karst regions present challenges for assessing and conserving stygobiont diversity and investigating their evolutionary history. We examined the karst‐obligate salamanders of the *Eurycea neotenes* species complex in the Edwards Plateau region of central Texas using population genomics data to address questions about population connectivity and the potential for gene exchange within the underlying aquifer system. The *E. neotenes* species complex has historically been divided into three nominal species, but their status, and spatial extent of species ranges, have remained uncertain. We discovered evidence of extensive admixture among species within the complex and with adjacent lineages. We observed relatively low levels of differentiation among all sampling localities which supports the hypothesis of recent divergence. Nominal taxonomy, aquifer region and geography each accounted for a modest amount of the overall population genomic variation; however, these predictors were largely confounded and difficult to disentangle. Importantly, current taxonomy of the three nominal species does not reflect the admixture apparent in clustering analyses. Inference of migration events revealed a complex pattern of gene exchange, suggesting that *Eurycea* salamanders have a dynamic history of dispersal through the aquifer system. These results highlight the need for greater understanding of how stygobiont populations are connected via dispersal and gene exchange through karst aquifers. These results also highlight the applicability of population genomics data as a powerful lever for investigating connectivity among populations in systems where direct detection of dispersal paths is difficult, as in underground, aquatic systems.

## Graphical abstract

Population genomics analyses of the karst‐inhabiting *E. neotenes* complex of salamanders reveals low levels of differentiation and substantial admixture among the three nominal species in the complex. The history of these populations includes recent divergence and gene exchange through aquifers.

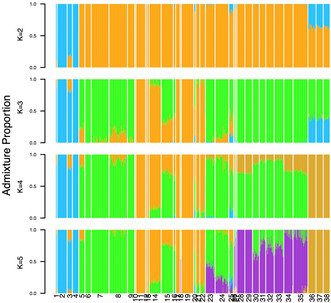

## 1 Introduction

Limestone karst landforms are considered “islands” of terrestrial and aquatic habitats that often exhibit extraordinary biodiversity (Barr Jr and Holsinger 1985; Clements et al. 2006; Hutchins et al. 2021; Longley 1981). Within karst islands, cave and aquifer systems form a complex, and often human‐inaccessible, subterranean landscape for both terrestrial and aquatic organisms. The subterranean complexity contributes to species richness through dispersal limitation, multiple colonizations, opportunities for vicariance, and diverse, sometimes extreme habitats that foster the evolution of ecological, behavioral, and morphological specialization (Bendik et al. 2013; Culver, Pipan, and Schneider 2009).

Karst biotas include many endemic species with relatively narrow geographic ranges that are also sensitive to perturbations. Conservation of these ecosystems and their constituent species is sometimes hampered by a lack of knowledge about their biogeographic, evolutionary, and demographic histories. In particular: (1) The number of distinct lineages or species might be underestimated due to under sampling, insufficient taxonomic knowledge (e.g., Hutchins et al. 2021), or convergent or parallel evolution (Wiens, Chippindale, and Hillis 2003); alternatively, diversity can be overestimated due to conflicts between morphological and genetic data. (2) The extent of species ranges (or the boundaries between them) are often quite difficult to Edwards Plataue delineate given subterranean complexity and inaccessibility in karst regions. As a result of the complexity of subterranean habitat geography, (3) the conduits for population connectivity (via gene flow) can be difficult to predict and might differ among even closely related lineages with divergent adaptive histories (Katz, Taylor, and Davis 2018). Furthermore, the conduits for gene flow can be dynamic depending on aquifer recharge rates and water levels (Longley 1981). We confront these knowledge gaps with population genomics data from extensive sampling of the *Eurycea neotenes* salamander species complex in the central Edwards Plateau region of Texas, one of the most diverse karst systems (Longley 1981).

**FIGURE 1:**
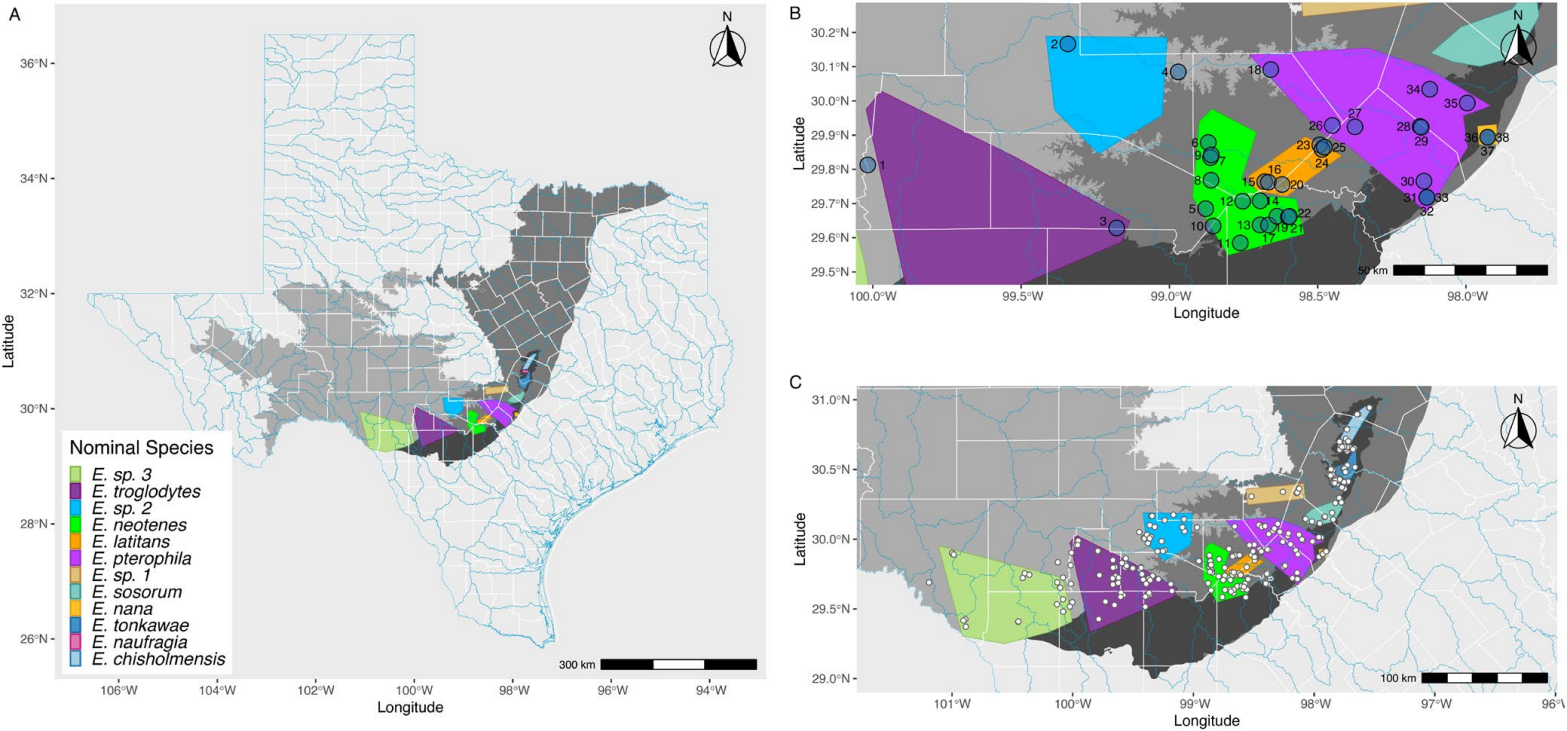
Map of the central Edwards Plateau region of Texas with *Eurycea* salamander locations, approximate ranges and sampling localities for this study indicated. (A) Major aquifers in the state of Texas and approximate ranges of nominal *Eurycea* lineages. Shading indicates specific components of the aquifer system: Edwards‐Trinity Aquifer (light gray), Trinity Aquifer (medium gray), Edwards Aquifer (dark gray). These aquifer components are variously connected and form the Edwards‐Trinity aquifer system. The approximate ranges of the nominal *Eurycea* lineages are indicated by colored polygons. (B) Focused map of sampling localities for this study. Locality numbers follow Table 1. (C) Approximate locations of *Eurycea* occurrences, including known (historical) localities, localities sampled by Devitt et al. (2019) and sampling localities for this study. Range information based on Devitt et al. (2019), Chippindale et al. (2000), Bendik et al. (2013), and personal observations.

The Edwards‐Trinity Aquifer system underlies the Edwards Plateau region of central Texas (Figure 1), an area of uplifted limestone that is home to a large number of cave‐ and spring‐associated species. The rich stygobiont diversity of the aquifers is increasingly impacted by encroaching human population density in the area. Human impacts include altered recharge patterns (Lamichhane and Shakya 2019), higher levels of nutrients (Boyer et al. 2002) and contaminants (Diaz et al. 2020), as well as lowered water tables in parts of the aquifer (Green et al. 2011). These anthropogenic stressors are exacerbated by climate change and have the potential to profoundly affect regional and local biodiversity.

Among the Edwards Plateau region stygobionts impacted by these stressors are up to 14 species of endemic, fully aquatic, neotenic salamanders of the genus *Eurycea* (Plethodontidae: Hemidactyliinae) (Devitt et al. 2019) that inhabit springs and caves throughout the Edwards Plateau (Figure 1). Ten of these species are listed as threatened or endangered at the federal or state level (https://ecos.fws.gov, https://tpwd.texas.gov/). These salamanders have adapted to the aquifer habitats since their colonization of the aquifers and are quite specialized. Some of the species are adapted to deep aquifer habitats and exhibit classic convergent troglobitic (subsurface) morphologies that include loss of eyes, depigmentation, and elongation of limbs and skull, while other species reside in springs and caves and exhibit surface‐dwelling morphologies, retaining eyes and pigmentation (Devitt et al. 2019; Hillis et al. 2001). The *Eurycea* salamanders of the Edwards Plateau are specialized stygobionts that are relatively intolerant of fluctuations in water temperature (Barr and Babbitt 2002; Crow et al. 2016) and quality (Bowles, Sanders, and Hansen 2006). These salamanders are completely reliant upon groundwater, which makes the encroachment of urban areas and climate change increasing conservation concerns (Bendik et al. 2014; Diaz et al. 2020).

Despite considerable research into the phylogeny and population genetic structure of spring‐associated salamanders in central Texas (e.g., Bendik et al. 2013; Chippindale et al. 2000; Devitt et al. 2019; Hillis et al. 2001; Lucas et al. 2009), uncertainty remains regarding the boundaries between, and the extent of gene exchange among, the lineages. We focused on a segment of *Eurycea* diversity in the southeastern Edwards Plateau that is home to several closely related nominal species in the *E. neotenes* species complex (Chippindale et al. 2000; Wiens, Chippindale, and Hillis 2003; Devitt et al. 2019). Historically, four species have been recognized within the region: the Texas salamander ( *E. neotenes* ; Bishop and Wright 1937), the Cascade Cavern's salamander ( *E. latitans* ; Smith and Potter 1946), the Fern Bank Salamander ( *E. pterophila* ; Burger, Smith, and Potter Jr 1950), and the Comal Blind Salamander ( *E. tridentifera* ; Mitchell and Reddell 1965), however, Devitt et al. (2019) synonymized *E. tridentifera* within *E. latitans* . These nominal species are largely surface forms, though some localities include variation that tends toward the subterranean forms (Bendik et al. 2013). This group has a complicated taxonomic history with morphological (Sweet 1984) and genetic (Bendik et al. 2013; Devitt et al. 2019) evidence of incomplete isolation and ongoing gene flow.

Devitt et al. (2019) surveyed genomic variation across the diversity of central Texas *Eurycea* salamanders, inclusive of the *E. neotenes* complex, concluding that the number of distinct lineages or species in the *E. neotenes* complex was lower than historically recognized. Here, we build on Devitt et al. (2019)'s call for further investigation to pursue questions of the nature and extent of admixture in this group. The uncertainty about these species and the extent of gene flow among them is due in part to the apparent recent evolution of the group and relatively limited sampling throughout the ranges of these salamanders (Chippindale et al. 2000; Devitt et al. 2019). We focus specifically on the *E. neotenes* complex in order to identify in detail the geographic ranges of lineages and admixture among them. We also specifically address questions about the nature of observed admixture and the possible history of gene flow within this group of salamanders. The results of these investigations shed light on the evolutionary history of these salamanders, the potential for gene flow through aquifers in the region and provide concrete information for the detailed management of these salamanders at a fine spatial resolution which is critical as human population density and water consumption increase dramatically in the region.

Here, we sampled the *E. neotenes* complex of salamanders from the Guadalupe and San Antonio river drainages in central Texas. We sampled extensively with respect to both geography and samples per locality (Table 1, Figure 1) and generated population genomic data to address the following questions: (1) Do patterns of population genomic variation support the nominal taxonomy of three distinct lineages of surface salamanders corresponding to *E. pterophila* , *E. neotenes* and *E. latitans* ? (2) How differentiated are populations and lineages of these salamanders? and (3) to what extent have populations or lineages of these salamanders been connected by historical or contemporary gene flow? The answers to these questions provide critical insights for determining the ongoing conservation status of these salamanders, guiding their management, and furthering our understanding of how organisms utilize karst habitats.

**TABLE 1:** Sampling details.

Locality number	Locality name	*n*	Nominal taxonomy	Major aquifer
1	Nueces 335	2	*E. troglodytes*	Edwards‐Trinity
2	Fessenden Springs	23	*E*. sp. 2	Edwards‐Trinity
3	Hill Country SNA	11	*E. troglodytes*	Trinity
4	Western Kerr	14	*E*. sp. 2	Edwards‐Trinity
5	Osborn Spring	13	*E. neotenes*	Trinity
6	Possum Creek Spring	14	*E. neotenes*	Trinity
7	Brown Ranch	42	*E. neotenes*	Trinity
8	Albert and Bessie Kronkosky SNA	44	*E. neotenes*	Trinity
9	Salamander Spring	16	*E. neotenes*	Trinity
10	Pecan Springs Cave	1	*E. neotenes*	Trinity
11	Government Canyon	22	*E. neotenes*	Edwards
12	Mueller's Ranch	2	*E. neotenes*	Trinity
13	Helotes Creek Spring	3	*E. neotenes*	Trinity
14	Maverick Ranch	28	*E. neotenes*	Trinity
15	Cascade Caverns	28	*E. latitans*	Trinity
16	Pfeiffer Ranch	4	*E. latitans*	Trinity
17	Leahs Spring	9	*E. neotenes*	Trinity
18	Peavey's Spring	1	*E. latitans*	Edwards‐Trinity
19	Leon Springs	29	*E. neotenes*	Trinity
20	Badweather Pit	3	*E. latitans*	Trinity
21	Camp Bullis Stealth	8	*E. neotenes*	Trinity
22	Camp Bullis Sharon	12	*E. neotenes*	Trinity
23	Guadalupe River State Park	22	*E. latitans*	Trinity
24	Honey Creek SNA Springs	32	*E. latitans*	Trinity
25	Preserve Cave	10	*E. latitans*	Trinity
26	Sattler's Deep Pit	2	*E. latitans*	Trinity
27	Rebecca Spring	2	*E. latitans*	Trinity
28	Devil's Backbone	18	*E. pterophila*	Edwards
29	Ott's Spring	17	*E. pterophila*	Trinity
30	Hueco Springs	15	*E. pterophila*	Edwards
31	Comal Spr Run One	14	*E. pterophila*	Edwards
32	Comal Spr Run Three	19	*E. pterophila*	Edwards
33	Comal Spr Spring Island	22	*E. pterophila*	Edwards
34	Jacobs Well	21	*E. pterophila*	Trinity
35	Fern Bank	35	*E. pterophila*	Trinity
36	San Marcos below dam	18	*E. nana*	Edwards
37	San Marcos Diversion	16	*E. nana*	Edwards
38	San Marcos Hotel	15	*E. nana*	Edwards
	Total	607		

*Note:* Locality names and samples sizes (*n*) for individuals genotyped for each locality.

## 2 Materials and Methods

### 2.1 DNA Sequencing and Data Collection

Sampling for this investigation included extensive collections of the focal *E. neotenes* complex (including the nominal taxa *E. pterophila* , *E. neotenes*, and *E. latitans* ). We also included samples of *E. nana* , which is closely related to the *E. neotenes* complex (Bendik et al. 2013; Chippindale et al. 2000; Devitt et al. 2019) and samples from further west of the focal area for comparison. These western samples are historically assignable to *E. troglodytes* and “*E*. sp. 2”, an undescribed species identified by Devitt et al. (2019) who recognized significant differentiation within *E. troglodytes* (Table 1 and Table S1). Tissues from tail clips were collected from 374 wild‐caught individual salamanders from 2019 to 2021 under Texas Parks and Wildlife Department permit SPR‐0111‐003 and tail tissue was donated to Texas State University. Collecting instruments were sterilized between all individuals and sampling localities. Sampling was augmented with archived material from three sources: 17 tissue samples from museum specimens housed at the Texas Natural History Collections, Texas Memorial Museum at The University of Texas at Austin, Austin, Texas, collected from 1990 to 1994, DNA samples from 10 individuals from Preserve Cave donated by Tom Devitt, and DNA samples from 242 salamanders generated for a previous study from individuals collected from 2004 and 2005 (Lucas et al. 2009) (Table 1 and Table S1, Figure 1). All tissue samples were preserved in 95% EtOH. Samples from Lucas et al. (2009) were extracted using the Promega Wizard SV Genomic DNA Purification System and the Gentra Systems Puregene DNA Purification kits. DNA from all other samples was extracted using the DNeasy Blood and Tissue Kit (Qiagen Inc., Alameda, CA, USA).

Following the methods of Parchman et al. (2012) and Gompert et al. (2014), we constructed a reduced representation genomic library for each of 643 salamanders. For these libraries, genomic DNA was digested with the EcoR1 and Mse1 restriction enzymes. To the resulting fragments, Illumina adaptors with unique 8–10 bp individual multiplex identifier (MID) sequences were ligated. Fragments were then amplified with two rounds of PCR using iProof high‐fidelity polymerase (BioRad Inc.). Amplicons from these PCR reactions were then pooled. Fragments between 300 and 450 bp were selected using a BluePippin (Sage Science Inc., Beverly, MA, USA) and the resulting fragments were sequenced on two lanes of Illumina Novaseq 6000 (single read, 100 bp) at the University of Texas Austin Genomic Sequencing and Analysis Facility (Austin, Texas, USA).

### 2.2 Assembly, Alignment, and Variant Calling

From the resulting sequence reads, PhiX reads were removed by assembly to the PhiX genome using bowtie version 1.1.2 (Langmead et al. 2009). Custom scripts were used to remove MIDs from each read and to filter short reads and reads that contained Mse1 adapter sequence. The resulting sequence reads were written to individual files in fastq format (median = 1,042,576 sequence reads per individual). 36 individuals each produced fewer than 400,000 reads and were excluded from further analyses. Excluded individuals originated from 21 distinct localities and included a maximum of four individuals from any one locality. One specimen from the Texas Natural History Collections (from Pfeiffer Ranch (16); we refer to specific localities by name and corresponding number following Table 1 in parentheses) was among the excluded individuals. The reads from the remaining 607 individuals (Table 1, Figure 1, Table S1) were filtered and clustered using the strategy described in the dDocent variant calling pipeline (Puritz, Hollenbeck, and Gold 2014) and CD‐hit (Fu et al. 2012). We ignored sequence reads with less than four copies per individual and shared among less than four individuals. The filtered reads were assembled with a homology threshold of 80% (other thresholds from 80% to 95% were investigated but produced very similar assemblies, data not presented). The consensus reads from this *de novo* assembly were used as the basis for a reference‐based alignment of all reads using the *aln* and *samse* algorithms of the Burrows‐Wheeler Aligner (bwa version: 0.7.12) (Li and Durbin 2009) with a seed length of 20 and up to four mismatches allowed.

Following assembly and alignment, we identified variable sites, or polymorphic single nucleotide sites (SNPs), with bcftools version 1.9 (Li et al. 2009) using the *mpileup* and *call* commands, ignoring indels and retaining variable sites if the posterior probability that the nucleotide was invariant was < 0.05. We performed additional filtering on the Variant Call Format file using custom scripts to exclude variable sites with sequence depth less than 607 reads (an average of one read per site per individual, 1×) and greater than 47,246 reads (equal to the mean sequence depth across sites plus two standard deviations; this filter is designed to remove potentially paralogous reads), less than at least 20 reads of the alternative allele, mapping quality less than 30, an absolute value of the mapping quality rank sum test greater than 1.96, an absolute value of the read position rank sum test greater than 1.96, an absolute value of the base quality rank sum test greater than 1.96, minor allele frequency less than 0.05, or missing data for more than 303 individuals (50% of individuals). One variable site per contig was chosen randomly and retained to maximize independence among loci. The genotype likelihoods from the resulting variable sites (SNP loci) were updated using the Bayesian admixture algorithm entropy (Gompert et al. 2014; Shastry et al. 2021) to provide posterior genotype probabilities for analyses.

### 2.3 Analyses

To answer our first two questions pertaining to the organization of genetic variation, we estimated genotypes, allele frequencies, and admixture proportions and used these to illustrate patterns of genomic variation. We estimated admixture proportions for each individual, allele frequencies for each cluster (population), and posterior genotype probabilities for all loci for all individuals using the Bayesian admixture algorithm entropy (Gompert et al. 2014; Shastry et al. 2021). entropy also facilitates model evaluation using standard Bayesian model diagnostics. A series of models were fit to the data varying the number of clusters or populations (*k*) from 2 to 15. Two MCMC simulations (chains) of 105,000 steps, with a burnin of 5000 steps, retaining every 10th step (total of 10,000 steps for each chain), were run for each *k*. We calculated Gelman and Rubin's convergence diagnostic and effective sample sizes (Brooks and Gelman 1998; Gelman and Rubin 1992) for each chain with the package coda version 0.19–1 in R (Plummer et al. 2006; R Core Team 2022) to assess model performance and check that the models reached a stable sampling distribution. We rejected model results when the mean Gelman and Rubin's convergence diagnostic across individual values of the admixture proportion, *q*, was greater than 1.1 or when increasing *k* did not result in a recognizable cluster. Models for *k* = 2–5 satisfied these criteria. Attempts to employ more MCMC steps, longer burnin, and different thinning strategies failed to rescue models with higher numbers of clusters (data not presented). Posterior estimates of genotypes were then averaged across all MCMC steps for models *k* = 2–5. Patterns of variation among individuals were illustrated by ordination of multilocus genotypes using principal component analysis (PCA), and patterns of admixture were illustrated with barplots, all performed in R (R Core Team 2022). To quantify differentiation among sample sites, we calculated genome‐average Nei's *G*_ST_ (Nei 1973). *G*_ST_ is an analog of *F*_ST_ that is generally applicable and appropriate for pairwise comparisons of population samples, and calculated as $G_{\mathrm{ST}}=\frac{\frac{1}{n}\sum_{n}\left(H_{T}-H_{S}\right)}{\frac{1}{n}\sum_{n}\left(H_{T}\right)}$ for all pairwise combinations of sites, which we refer to as *F*_ST_ hereafter. *F*_ST_ was calculated as the average across all loci and 1000 bootstrap resamples were used to calculate 95% confidence intervals.

We then used samtools version 0.1.9 to calculate two measures of genetic diversity for all aligned nucleotide sites for each of the 29 localities with samples of ≥ 8 individuals. We estimated Watterson's *θ* (based on the number of segregating sites) and Tajima's *π* (nucleotide diversity or heterozygosity) using the expectation–maximization algorithm with 20 iterations (Li 2011), which was sufficient for values of both metrics to converge for all localities.

To specifically address our first question regarding whether genomic variation is organized according to the nominal taxonomy (i.e., three nominal species in the *E. neotenes* complex), we employed two approaches: variance partitioning using redundancy analysis (RDA) (Capblancq and Forester 2021) and a Bayesian linear mixed modeling approach (Gompert et al. 2014). First, we used RDA to partition genetic variance using predictors representing the historical nominal taxonomy plus features of geology (aquifer zones) as a comparison, and geographical space. These analyses were performed in R (R Core Team 2022). To the best of our ability, we assigned localities to a nominal species based on historical descriptions and previous taxonomic studies that employed molecular marker data (Bendik et al. 2013; Chippindale et al. 2000; Devitt et al. 2019). It should be noted that because many of our sampling localities have not been sampled previously there is a substantial possibility for error in these assignments. These nominal species designations (Table 1) were used as predictors of genomic variation in RDAs. Similarly, we used major aquifer designation for each locality as predictors (Table 1). The localities included sites located in the Edwards—Trinity Aquifer in the western portion of the Edwards Plateau, sites in the Trinity Aquifer in the northern regions of our sampling area, and sites in the Balcones Fault Zone of the Edwards Aquifer along the southeastern edge of our sampling area (Table 1, Figure 1). We accounted for geography by using Moran's Eigenvector Mapping functions (MEMs) which describe spatial autocorrelation of our sampling sites at multiple scales (Borcard and Legendre 2002; Borcard et al. 2004; Dray, Legendre, and Peres‐Neto 2006; Forester et al. 2018). MEMs were calculated using the *dbmem* function from the adespatial package. We converted the posterior genotype matrix (described above) into a Euclidean distance matrix among individuals using the *vegdist* function in the vegan package. (This does not alter results of any of the analyses but does reduce computational time.) We performed RDA using nominal taxonomy, aquifer, and MEMs as predictors of genomic variation using the *dbrda* function from the vegan package followed by permutational ANOVA. We partitioned variance for each of the predictors using the *varpart* function in the vegan package.

In a second method for addressing whether genetic variation was organized along nominal taxonomic divisions or major aquifers, we constructed a Bayesian mixed model regression to compare geographic distance, “taxonomic” distance and “aquifer” distance as predictors of population differentiation (Gompert et al. 2014). Specifically, we used this model to ask whether population differentiation was best explained by geography, nominal taxonomy, major aquifer, or combinations of these predictors. The regression model accounts for the correlated error structure in pairwise distance measures (genetic, geographic, taxonomic, and aquifer) by incorporating random effects for each population that represent mean population‐specific deviations in the distance matrices following Clarke, Rothery, and Raybould (2002). Thus,

$$Y_{\mathrm{ij}}=\beta_{0}+\beta_{\mathrm{GEO}}X_{\mathrm{ij}}^{\mathrm{GEO}}+\beta_{\mathrm{TAX}}X_{\mathrm{ij}}^{\mathrm{TAX}}+\beta_{\mathrm{AQU}}X_{\mathrm{ij}}^{\mathrm{AQU}}+\alpha_{i}+\alpha_{j}+\epsilon_{\mathrm{ij}}$$

where *Y*_*ij*_ is pairwise genetic distance between populations *i* and *j*, $X_{\mathrm{ij}}^{\mathrm{GEO}}$ is the corresponding geographic distance, $X_{\mathrm{ij}}^{\mathrm{TAX}}$ indicates taxonomic identity (with 1 when populations *i* and *j* are from different nominal taxa and 0 when from the same nominal taxon), and $X_{\mathrm{ij}}^{\mathrm{AQU}}$ indicates major aquifer identity (with 1 when populations *i* and *j* are located in different major aquifers and 0 when from the same major aquifer) (Table 1). The *β*'s are fixed effect regression coefficients. The *α* terms represent random effects for individual populations (Clarke, Rothery, and Raybould 2002) and *ϵ*_*ij*_ is the error. For genetic distances, we used pairwise *F*_ST_/(1 − *F*_ST_) (Rousset 1997), (*F*_ST_ calculated as described above and for all localities with *n* ≥ 8). Pairwise genetic distances were log transformed and centered. The geographic distance matrix comprised great circle distances between localities expressed in meters which were log transformed, centered, and scaled. We used uninformative Gaussian priors for the regression coefficients (*β*'s) (*μ* = 0, *σ* ^2^ = 1000) and hierarchical Gaussian priors for the population random effects (*α*'s) (*μ* = 0, ${\sigma^{2}=\sigma }_{\mathrm{pop}}^{2}$) and uninformative gamma priors for the random effects and residual variances (as reciprocals) (*α* = 1, *β* = 0.01). Models were implemented in r using rjags with MCMC run in jags (Plummer 2003). All models included three MCMC chains of 10,000 iterations, a burnin of 2000 iterations and thinning every five steps. Models were constructed for the entire dataset of localities with *n* ≥ 8 (29 localities) and for a “focal” dataset of localities assigned to the *E. neotenes* complex (23 localities). We fit full models with terms for geography, taxonomy, and aquifer, plus models with two‐way combinations of predictors, and models with single predictors. To assess predictive accuracy of all models and to facilitate model comparisons, we used approximate leave‐one‐out cross‐validation (LOO‐CV) using Pareto‐smoothed importance sampling (PSIS) (Vehtari, Gelman, and Gabry 2017; Vehtari et al. 2024) to calculate an information criterion statistic (LOOIC). Cross‐validation was performed using the *loo* function in the loo package in R (Vehtari, Gelman, and Gabry 2017). The LOOIC value is the estimated pointwise predictive densities from the leave‐one‐out cross‐validation transformed to the scale of deviance. Differences in LOOIC were calculated comparing models to the model with the lowest LOOIC score and standard errors of the differences were calculated on which models can be compared (McElreath 2020). In addition, we calculated and present a Bayesian estimate of R‐squared for every model as an indicator of model performance (Gelman et al. 2019).

Our third question regarding the possibility of gene exchange during the history of these populations presents challenges because observed admixture could result from incomplete lineage sorting (ancestral polymorphism), historical gene flow, contemporary gene flow, or combinations of these (e.g., Holder, Anderson, and Holloway 2001; Joly, McLenachan, and Lockhart 2009; Machado et al. 2002; Sang and Zhong 2000). It should be noted that many methods for inference of gene flow, including those used here, cannot readily discriminate between secondary contact with gene flow versus gene flow during divergence (Endler 1977; Pinho and Hey 2010). Given these difficulties, we employed three analyses with different approaches in an attempt to understand the history of gene exchange: We first used the methods of Pickrell and Pritchard (2012) as implemented in treemix version 1.12. Allele frequencies from all focal sampling localities with sample sizes of at least eight individuals were used to estimate the hypothesized population graph representing the evolutionary history of the sampled populations assuming no migration or admixture (*m* = 0). The sample from Fessenden Springs (2) was designated as the outgroup. We then sequentially evaluated the contribution of additional migration nodes (admixture events, *m* = 1–10). Each model of *m* migration nodes was replicated with 1000 runs and the proportion of the total explained variance in allele frequencies was calculated for each replicate using a custom Rscript written by D. Card (https://github.com/darencard/RADpipe/blob/master/treemixVarianceExplained.R). The asymptote in the median proportion of variance explained across replicates for each *m* was used as a guide to determine whether adding migration events improved model fit. Population graphs for *m* = 0 and for *m* with the highest median variance explained were plotted using the R package ggtree (Yu et al. 2017).

In a second analysis of gene exchange, we calculated *f*_3_ statistics (Patterson et al. 2012; Reich et al. 2009) with the threepop program from treemix version 1.12. *f*_3_ statistics specifically test the hypothesis that the evolution of sampled populations conforms to the expectations of a bifurcating model (i.e., the null hypothesis of no history of gene exchange). The test is conducted for sets of three populations, two potential source populations and a target population. Significantly negative *f*_3_ statistics indicate a departure from a strictly bifurcating evolutionary history for the three populations due to population admixture in the target population. We calculated *f*_3_ statistics for all sampling localities included in the treemix analyses described above.

As a complement to the above analyses designed to detect gene flow, we used eems (version 0.0.0.9000) (Petkova, Novembre, and Stephens 2016) to estimate an effective migration surface which provides a picture of the geography of departures from patterns of isolation‐by‐distance (IBD). This effective migration surface identifies areas with low or high gene flow relative to a stepping‐stone model. Our expectation is that gene flow among localities and lineages of *Eurycea* salamanders should appear as departures from the IBD expectation such that the effective migration surface will illustrate the presence of barriers to and/or corridors of gene flow. If, on the other hand, the history of divergence in these salamanders is one of primary divergence with little or no gene flow, we expect to observe few or no departures from IBD expectations. We estimated the effective migration surface assuming 700 demes and used three MCMC chains with 6 million iterations each, a burnin of 3 million iterations and a thinning interval or 10,000 iterations.

## 3 Results

**FIGURE 2:**
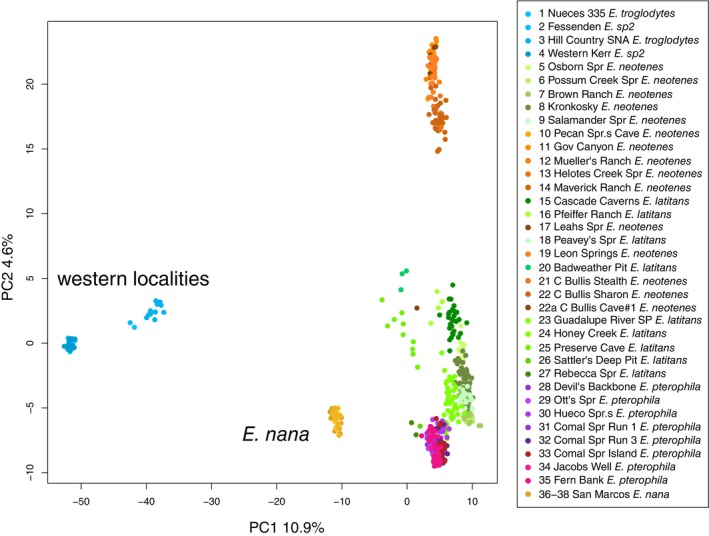
Principal component analysis of posterior genotype estimates summarizing variation among 607 *Eurycea* individuals based on 16,094 SNP loci. Each individual is represented by a point colored by locality (Table 1, Figure 1). The legend lists locality names and nominal species as inferred from previous research and geography. The “western localities” include localities 1–4 in Table 1 which are nominally *E. troglodytes* and *E. sp. 2*.

Sequencing produced 671,939,934 sequence reads with MIDs for 607 individuals (median = 1,072,524 reads per individual, standard error = 13,555.63, minimum reads = 420,551, maximum reads = 2,495,535). 42,274,123 reads were retained in the final assembly after filtering from which a SNP set of 16,094 loci was called. Mean sequence depth was 4.3 reads per individual per locus (standard error = 0.052) with an average of 26.4% missing data (i.e., no reads for an individual) per locus (standard error = 0.0012). Ordination of posterior genotype estimates from entropy showed differentiation of the western localities, *E. troglodytes* and *E*. sp.2 (*sensu* Devitt et al. 2019) from all other samples on PC axis 1 which explained ≈11% of the total genomic variation (Figure 2). Samples of *E. nana* were also differentiated from the focal *E. neotenes* complex, while clearly closely related. The focal *E. neotenes* complex separated into three less differentiated clusters on PC axis 2 which explained less than 5% of the variance. One group of individuals from localities in the southwestern portion of our sampling (localities 10–14, 17, 19, 21, 22, Table 1, Figure 1) formed a distinct cluster (colored variously orange in Figure 2). The other localities north (localities 5–9, 15, 16, 18, 20, 23–27 and colored green in Figure 2) and east (including 28–35 and colored purple/magenta in Figure 2) of the southwestern (orange) cluster are less differentiated from each other.

**FIGURE 3:**
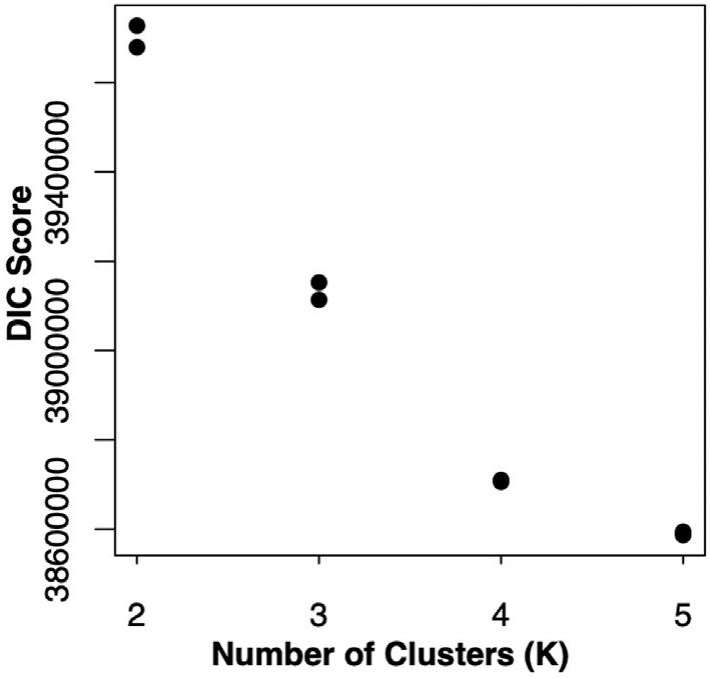
Deviance information criterion scores for MCMC chains for entropy models for *k* = 2–5.

**FIGURE 4:**
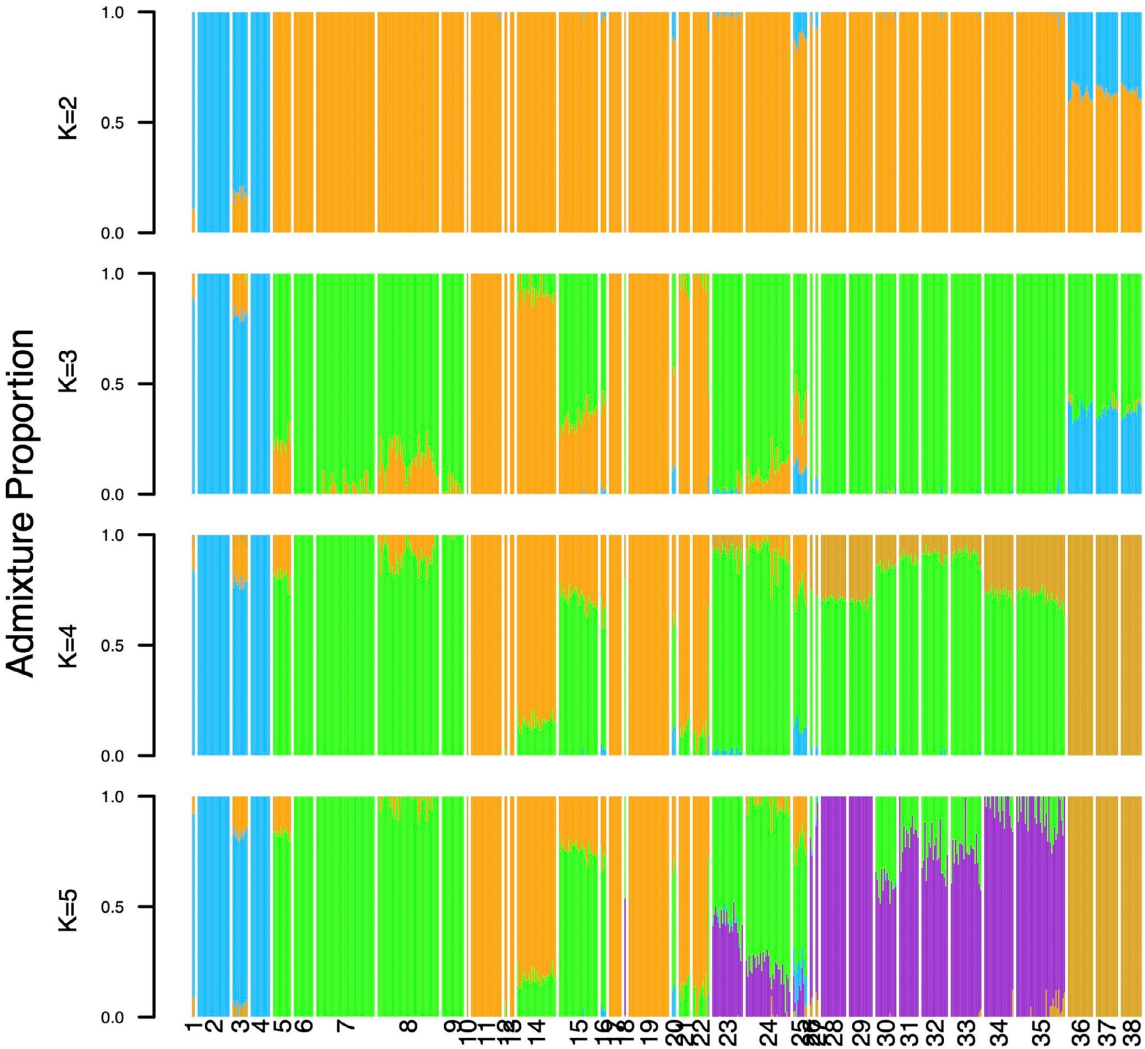
Admixture proportions for *k* = 2–5. Localities are oriented by longitude (west to east, Figures 1 and 5). Locality numbers follow Table 1. Localities 1 and 3 are referred to *E. troglodytes* , localities 2 and 4 are *E. sp 2* following Devitt et al. (2019), localities 5–35 include the *E. neotenes* species complex, and localities 36–38 are *E. nana* .

**FIGURE 5:**
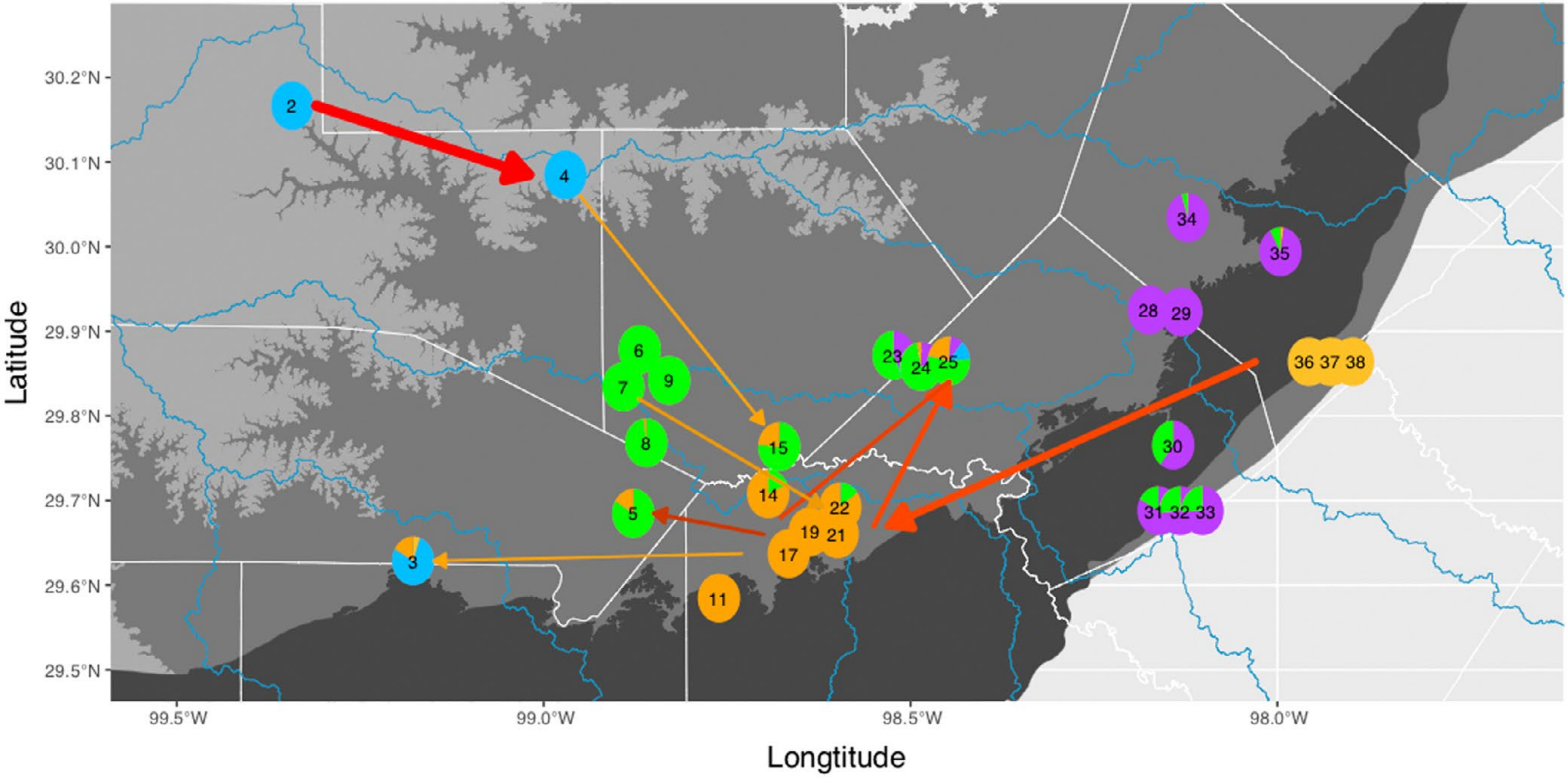
Admixture proportions per locality and migration events inferred from treemix. Pie diagrams represent proportional ancestry (*q*) for localities with at least eight sampled individuals based on entropy clustering for *k* = 5. Arrows show the direction of inferred migration events with color and magnitude as in Figure 13. Note that inferred migration events between localities cannot be interpreted as the actual paths of gene flow that occurred in the aquifers. Locality numbers follow Table 1.

Clustering analyses for *k* > 5 failed to meet our criteria for convergence thus we focus on results of *k* = 2–5. DIC scores were lowest for MCMC chains at *k* = 5 (Figure 3). Barplots of admixture proportions (Figure 4) mirror the apparent divisions in the PCA (Figure 2). The western localities (1–4) are differentiated first, followed by *E. nana* (36–38) at *k* = 4. For *k* = 5, the focal *E. neotenes* complex comprises three clusters as in the PCA. However, the clusters are not distinct and many localities show signs of admixture (Figures 4 and 5). Admixture is particularly evident at Cascade Caverns (15), the type locality for *E. latitans* (Smith and Potter 1946), Guadalupe River State Park (23), Hueco Springs (30), and all three Comal Springs sites (31–33). Honey Creek SNA (24), shows ancestry from all three groups within the *E. neotenes* complex, while Sattler's Deep Pit (26) and Rebecca Spring (27) show variable ancestry from four groups: from two of the *E. neotenes* complex (green and purple groups), from *E. nana* , and also from the western localities. Preserve Cave (25) shows ancestry from all five clusters. Further evidence of admixture is seen in the western localities, particularly Nueces 335 (1) and Hill Country State Natural Area (3) which share ancestry with the orange group (principally localities 11, 17 and 19) and *E. nana* . Some individuals from Jacobs Well (35) and Fern Bank (35) also exhibit *E. nana* ancestry.

**FIGURE 6:**
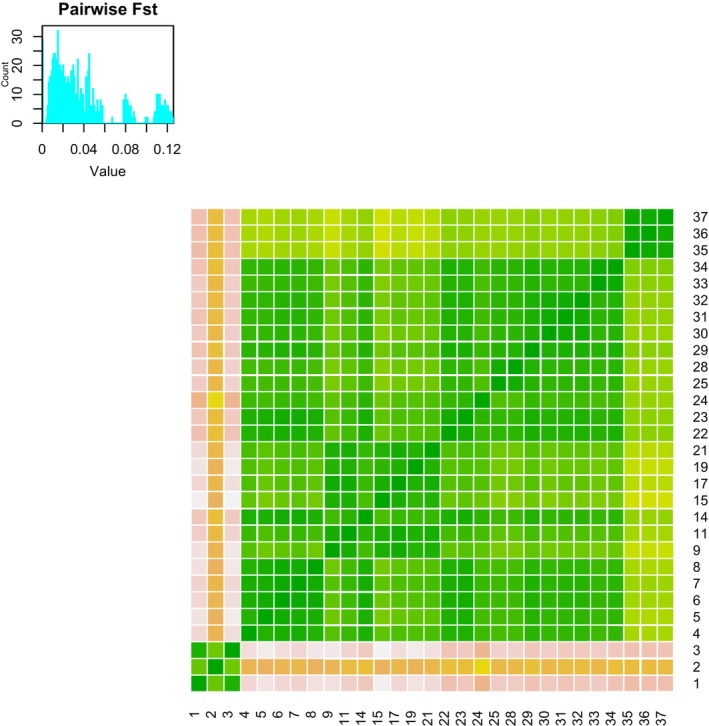
Heat map of pairwise *F*_ST_ values. Pairwise *F*_ST_ was calculated for all localities with *n* ≥ 8. Locality numbers follow Table 1.

Overall, levels of differentiation are low, especially within the *E. neotenes* complex. Pairwise *F*_ST_'s are very low (Tables S2–S5, Figure 6). The largest observed value (*F*_ST_ = 0.126, 95% confidence interval: 0.122–0.131) was between Western Kerr (4) and Leahs Springs (17). Excluding the western populations and *E. nana* (i.e., within the focal *E. neotenes* group), *F*_ST_'s ranged from 0.004 (0.004–0.004) (between Brown Ranch (7) and Albert and Bessie Kronkosky State Natural Area (8)) to 0.04 (0.038–0.042) between Leahs Springs (17) and Devil's Backbone (28), and 0.04 (0.039–0.042) between Leahs Springs (17) and Ott's Springs (29). These results are similar to those reported by Devitt et al. (2019) for this group (eastern *Blepsimogle*) and this level of differentiation is of the same magnitude as, or slightly higher than, population differentiation observed within another central Texas *Eurcyea*, *E. chisholmensis* (Nice et al. 2021).

**FIGURE 7:**
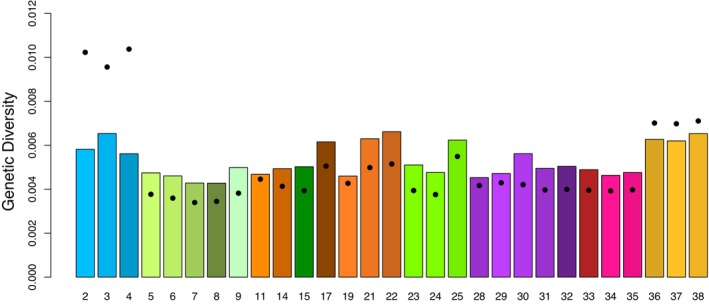
Genomic diversity estimated for *Eurycea* salamanders for all aligned sites from localities with at least eight sampled individuals. Nucleotide diversity as Tajima's *π* (expected heterozygosity) is plotted as bars. Watterson's *θ* is indicated as points for each site. Localities are arranged from west to east and locality numbers follow Table 1 and Figure 1.

Genetic diversity was slightly lower in the *E. neotenes* complex localities compared to *E. nana* or the western localities, especially for Watterson's *θ* (Figure 7). Diversity across all sampling localities was roughly a third to a half of the diversity measured by similar methods in *E. chisholmensis* (Nice et al. 2021). However, there were no obvious disparities in diversity across the *E. neotenes* complex.

**FIGURE 8:**
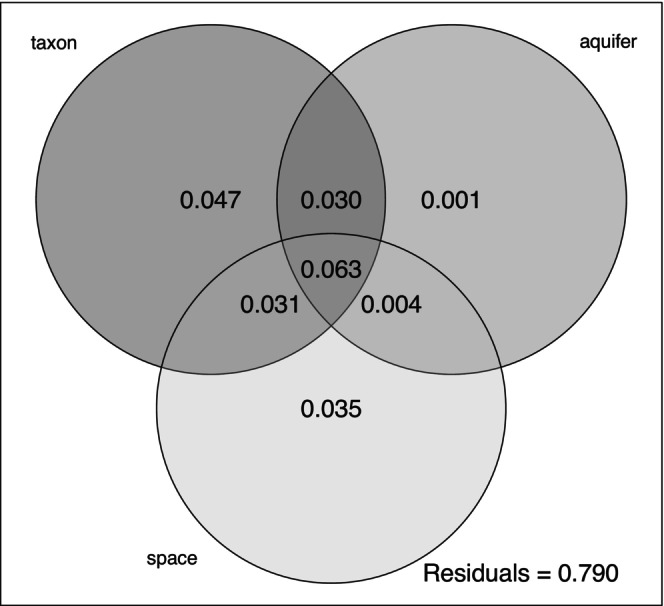
Venn diagram showing results of variance partitioning of the total genetic variance into components representing nominal taxonomy (taxon), major aquifer association (aquifer), and spatial autocorrelation (space) as captured by Moran's eigenvector mapping functions (MEMs). The full model explained 21% of the total variance.

Partitioning genotypic variation by nominal taxonomy and major aquifer (Table 1) and spatial autocorrelation using RDA revealed both low levels of variance explained and predictors that are confounded. Taxonomy alone (i.e., not controlling for spatial variation) explained 17.0% of total genotypic variation, while major aquifer alone explained 9.7%. Space, as captured by eight MEMs, all of which were significantly associated with genotypic variation, explained 13.3%. However, the combined model with nominal taxonomy, major aquifer and eight MEMs explained only 21% of the variance. In this combined model, taxonomy explained 4.7%, aquifer explained less than 1% and space accounted for 3.5% of the total genotypic variation (Figure 8), but the overlap of all three predictors (i.e., the variance explained by the combination of all three) was 6.3%, and two‐way entanglements between the predictors accounted for the remaining variance explained (Figure 8). In summary, spatial variation in the distribution of genetic variation in these populations is confounded with taxonomy and their distribution across major aquifers (Devitt et al. 2019).

**FIGURE 9:**
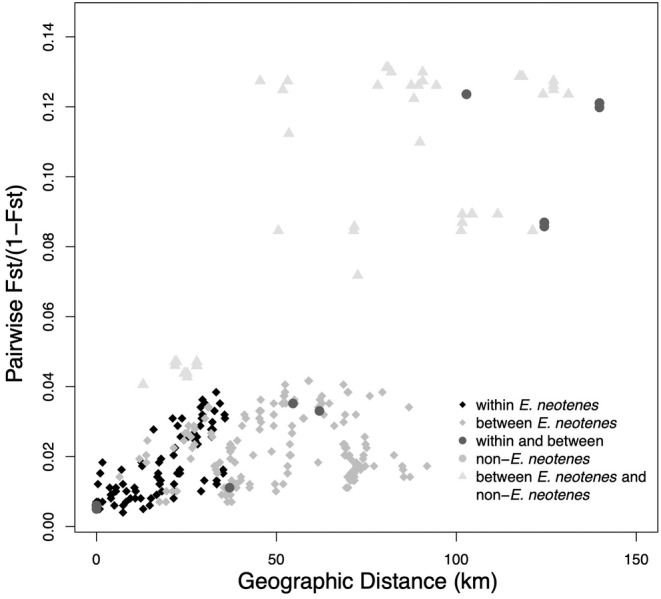
Relationship between geographical distance and genetic distance between localities as measured by *F*_ST_. Symbols and shading indicate different types of pairwise comparisons, including within the *E. neotenes* complex (i.e., comparisons between localities within each of the three nominal taxa), between the *E. neotenes* complex taxa (e.g., between *E. neotenes* and *E. pterophila* ), within and between non‐ *E. neotenes* taxa (e.g., within and between *E. troglodytes* and *E. nana* ) and between *E. neotenes* complex taxa and non‐ *E. neotenes* taxa (e.g., between *E. latitans* and *E. nana* ).

**FIGURE 10:**
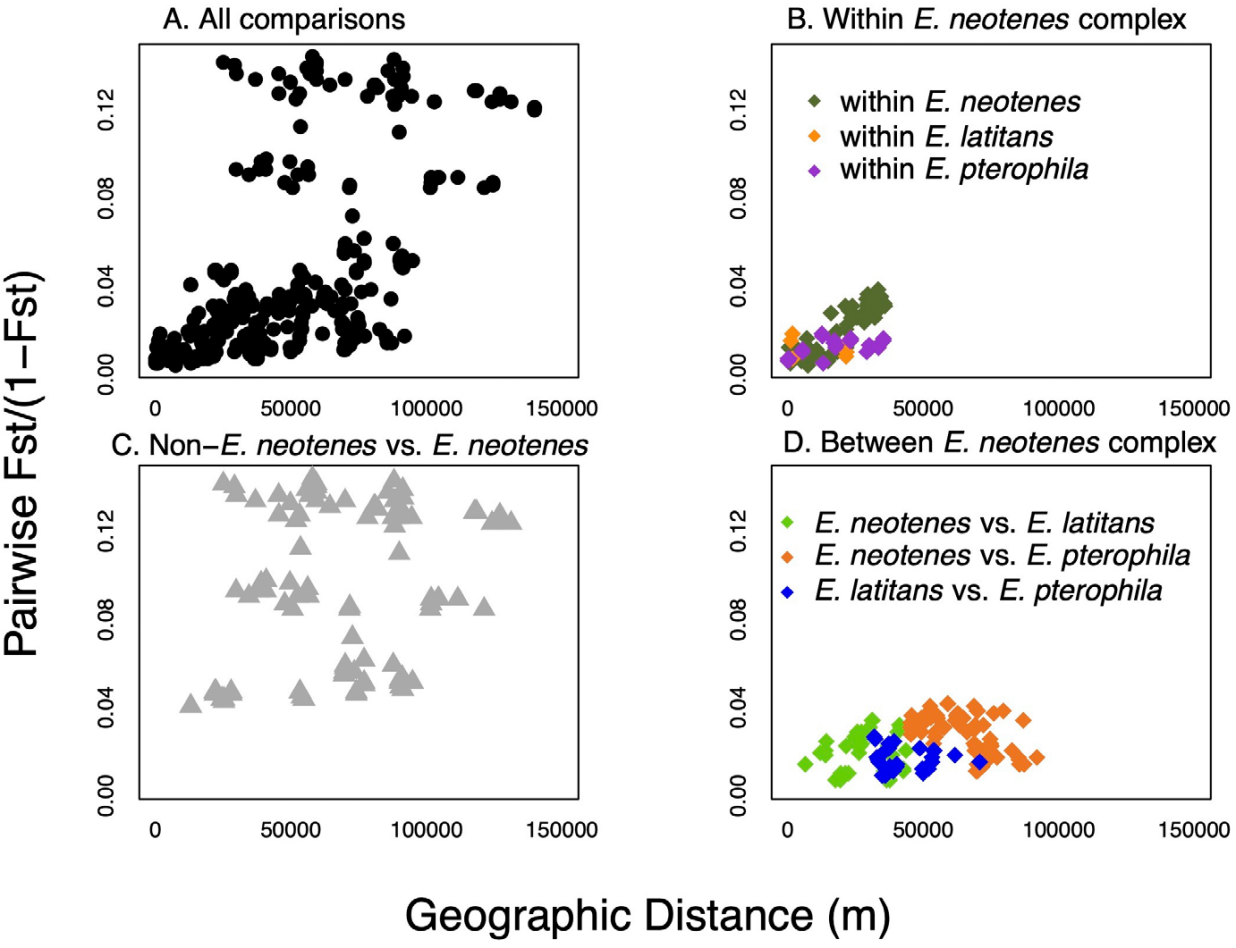
Patterns of genetic differentiation in relation to geographic distances. (A) Patterns of isolation by distance across all localities with *n* ≥ 8. (B) Patterns within the three nominal species in the *E. neotenes* complex. (C) Patterns in comparisons of *E. neotenes* complex taxa versus non‐ *E. neotenes* lineages. (D) Patterns in comparisons between *E. neotenes* complex taxa.

**FIGURE 11:**
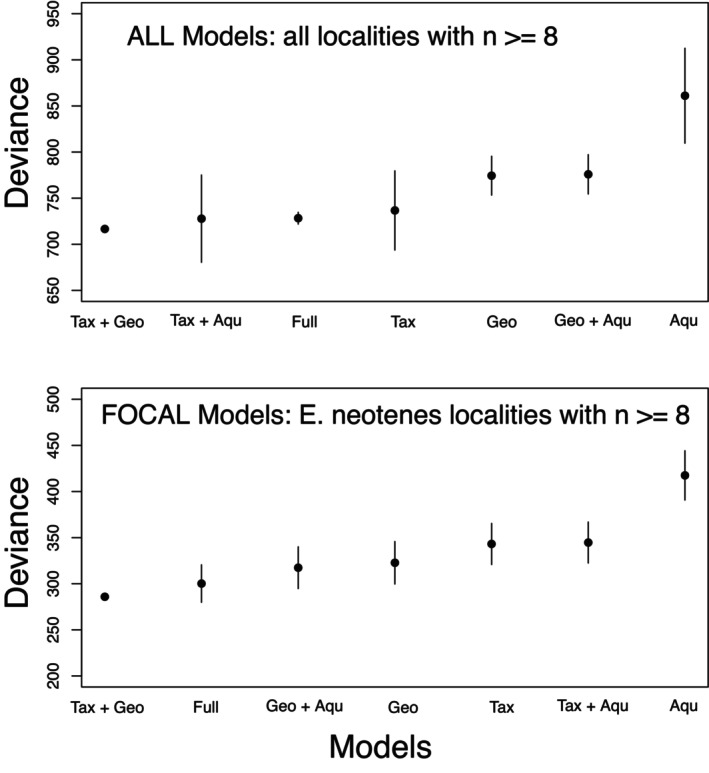
Comparison of Bayesian linear mixed models (BLMMs). LOOIC scores (points) and standard errors of the differences in LOOIC (compared to the model with lowest LOOIC deviance) (intervals) are plotted for models of all localities with *n* ≥ 8 (top panel), or for models of *E. neotenes* localities with *n* ≥ 8. Models are described along the *x*‐axis by the included predictor matrices following Table 2. Note that the first model (Tax + Geo) for both analyses has the highest predictive accuracy and thus the standard error of the difference is zero for these models.

The geographical signal detected in the examination of patterns of differentiation using RDA is further revealed by examining the relationship between pairwise differentiation and geographic distance (Figure 9). Within the three nominal focal species, differentiation increases mostly linearly with distance between localities (Figures 9 and 10). The Bayesian regression showed that more distant localities, localities classified as different taxa, and localities in different major aquifers were more genetically distinct (Table 2). Based on LOOIC, a model with terms for taxonomy and geography had the highest predictive accuracy for all localities as well as for the focal *E. neotenes* complex localities (Table 2). However, most other models were not different from the best model based on the standard error of the LOOIC differences (Table 2, Figure 11). Only models with aquifer distance as the sole predictor showed less predictive accuracy compared to other models (Table 2, Figure 11). Thus, as found with the RDA, genetic variation, in this case as pairwise genetic distances, appears to be organized along both geographic and taxonomic dimensions, and to a much lesser extent, major aquifer, all of which are confounded to some degree.

**TABLE 2:** Comparison of competing models predicting pairwise genetic distances between localities with taxonomic distance (Taxon), aquifer distance (Aquifer), and geographic distance (Geog)—see text for details.

Model	Coefficient	Mean (95% HDI)	LOOIC	LOOIC SE	dLOOIC	dLOOIC SE	*R* ^2^
All sites—Taxon and Geog	Intercept	−0.011 (−0.341, 0.296)	716.6	121.9	0	0	0.79
	*β*_TAX_	0.386 (0.248, 0.510)					
	*β*_GEO_	0.253 (0.200, 0.306)					
All sites—Taxon and Aquifer	Intercept	−0.004 (−0.313, 0.332)	727.8	87.2	11.2	47.4	0.74
	*β*_TAX_	0.705 (0.591, 0.827)					
	*β*_AQU_	0.188 (0.075, 0.308)					
All sites—Full	Intercept	0.006 (−0.348, 0.323)	728.3	126.7	11.7	6.4	0.79
	*β*_TAX_	0.384 (0.249, 0.508)					
	*β*_GEO_	0.251 (0.198, 0.308)					
	*β*_AQU_	0.011 (−0.109, 0.119)					
All sites—Taxon	Intercept	0.004 (−0.324, 0.362)	736.7	92.8	20.1	43.0	0.74
	*β*_TAX_	0.749 (0.627, 0.867)					
All sites—Geog	Intercept	0.013 (−0.321, 0.373)	774.4	137.6	57.8	21.2	0.77
	*β*_GEO_	0.342 (0.298, 0.386)					
All sites—Geog and Aquifer	Intercept	0.004 (−0.330, 0.344)	775.9	137.5	59.3	21.4	0.77
	*β*_GEO_	0.340 (0.291, 0.389)					
	*β*_AQU_	0.009 (−0.100, 0.125)					
All sites—Aquifer	Intercept	0.013 (−0.336, 0.380)	861.1	93.1	144.5	51.6	0.71
	*β*_AQU_	0.343 (0.217, 0.476)					
Focal sites—Taxon and Geog	Intercept	−0.001 (−0.186, 0.178)	285.8	32.3	0	0	0.62
	*β*_TAX_	0.219 (0.094, 0.335)					
	*β*_GEO_	0.204 (0.149, 0.263)					
Focal sites—Full	Intercept	−0.003 (−0.205, 0.197)	300.1	32.9	14.3	20.4	0.62
	*β*_TAX_	0.210 (0.089, 0.327)					
	*β*_GEO_	0.237 (0.1787, 0.295)					
	*β*_AQU_	−0.186 (−0.3237, 0.048)					
Focal sites—Geog and Aquifer	Intercept	0.002 (−0.193, 0.198)	317.3	35.0	31.5	22.7	0.62
	*β*_GEO_	0.294 (0.242, 0.346)					
	*β*_AQU_	−0.199 (−0.344, −0.056)					
Focal sites—Geog	Intercept	−0.004 (−0.184, 0.180)	322.7	35.7	36.9	23.1	0.59
	*β*_GEO_	0.262 (0.214, 0.308)					
Focal sites—Taxon	Intercept	0.000 (−0.194, 0.183)	343.1	29.4	57.3	22.4	0.63
	*β*_TAX_	0.463 (0.352, 0.571)					
Focal sites—Taxon and Aquifer	Intercept	−0.003 (−0.105, 0.176)	344.6	29.8	58.8	22.3	0.63
	*β*_TAX_	0.456 (0.348, 0.569)					
	*β*_AQU_	0.035 (−0.105, 0.176)					
Focal sites—Aquifer	Intercept	0.000 (−0.172, 0.183)	417.5	31.7	131.6	26.8	0.60
	*β*_AQU_	0.140 (−0.011, 0.289)					

*Note:* Shown are results of Bayesian linear mixed model regression ranked by Leave‐One‐Out Information Criteria (LOOIC) which is the expected log pointwise predictive density converted to deviance. Coefficients are posterior means with 95% highest density intervals in parentheses. Models with “All sites” include the 29 sites with *n* ≥ 8. Models with “Focal sites” include the 23 sites from the *E. neotenes* complex with *n* ≥ 8. LOOIC SE is the standard error of the LOOIC estimate, dLOOIC is the difference in LOOIC between a model and the model with the lowest LOOIC, dLOOIC SE is the standard error of the difference in LOOIC. Lastly, *R* ^2^ is the estimate of the Bayesian R‐squared.

**FIGURE 12:**
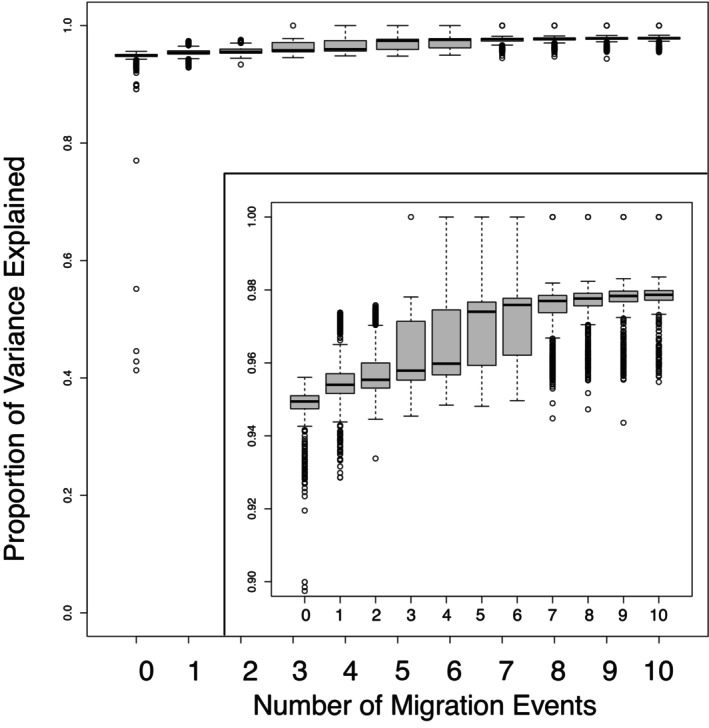
Boxplots of variance explained from treemix analyses of salamander localities with 0–10 migration events (*m* = 0–10). Boxplots show mean variance explained and variation from 1000 bootstrap replicates for each number of migration events, *m*. Inset shows magnified boxplots.

**FIGURE 13:**
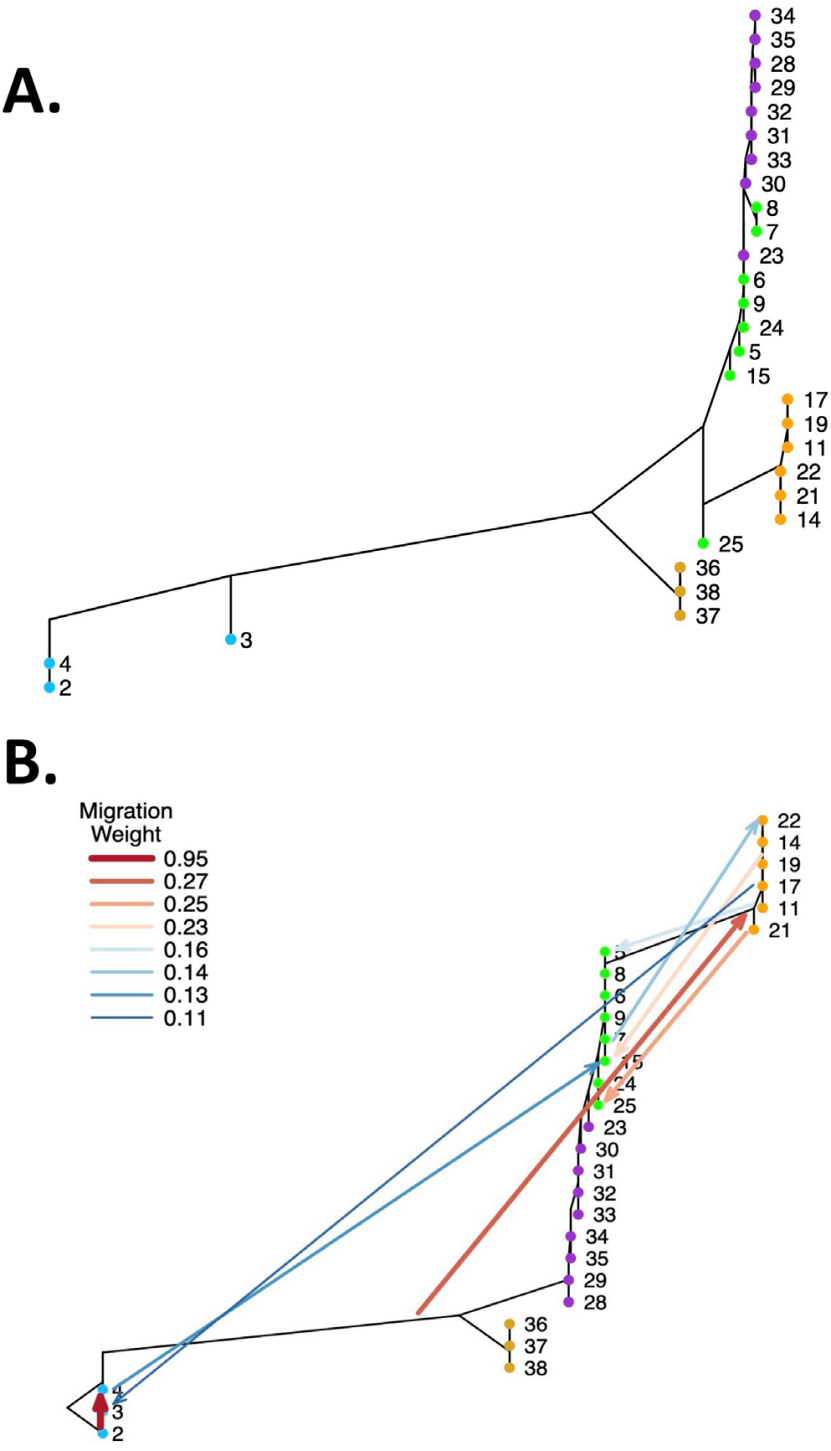
treemix results. (A) Drift tree (population graph with no migration, *m* = 0) of focal salamander localities with minimum sample sizes of at least eight individuals. Locality numbers follow Table 1. The Fessenden Springs site *E*. sp. 2, locality 2, serves as the outgroup. (B) Treemix population graph of focal salamander localities with eight migration events (*m* = 8). Locality numbers follow Table 1.

treemix analyses provided further evidence of gene exchange among populations. Adding migration events continued to improve the amount of variance explained until reaching an asymptote around eight migration events (Figure 12). The initial drift tree (population graph with no migration events, *m* = 0) has patterns similar to the PCA and barplots (Figure 13A) and explained considerable variance in allele frequencies. Adding migration events improved model fit though the increase in variance explained was small (Figure 12). The population graph incorporating eight migration events (*m* = 8) (Figure 13B) shows numerous migration events among *E. neotenes* complex populations, commonly involving the southwestern localities (localities 11, 14, 17, 19, 21, 22). There is also evidence of historical gene exchange between the western localities ( *E. troglodytes* and *E*. sp. 2) and *E. neotenes* complex populations and an historical gene flow event from *E. nana* to the southwestern localities. Many of these migration events parallel the patterns of admixture in the clustering analyses (Figure 4). Importantly, the direction of these migration events indicates multiple paths for gene flow (Figure 5) and that gene flow appears to have been relatively unrestricted in geographical space. Figures S1 and S2 present population graphs with seven (*m* = 7) and nine (*m* = 9) migration events.

Significantly negative *f*_3_ statistics were detected in more than 48% of the 10,962 three‐locality comparisons and included every locality as a target (i.e., receiving gene flow from the source populations) (Table S6). These results indicate that the history of the central Texas *Eurycea* is inconsistent with a bifurcating pattern of divergence and are an indication of a history of some gene exchange among localities. These results, in combination with the treemix results, suggest that admixture among localities has been a component of the history of these salamanders.

**FIGURE 14:**
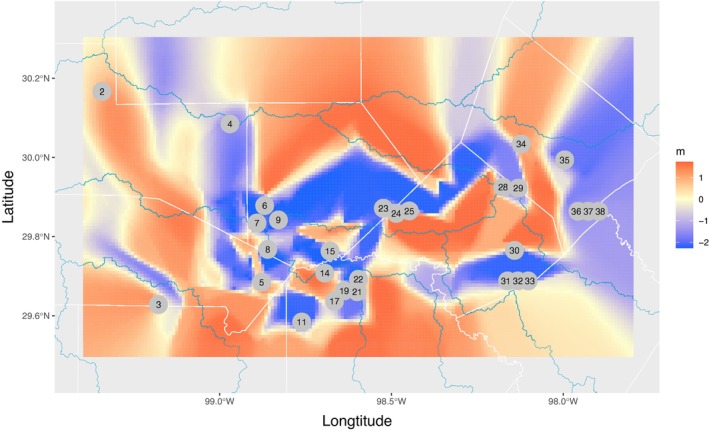
Estimated effective migration surface. The migration surface indicates departures from an isolation‐by‐distance, stepping‐stone model. Areas in red indicate higher migration (*m*) than expected; areas in blue indicate lower migration rates than expected. Only localities with *n* ≥ 8 are included in this analysis. Locality numbers follow Table 1.

The estimated effective migration surface revealed distinct areas where gene flow was higher or lower than expected under a two‐dimensional stepping‐stone model (Figure 14). A corridor (an area of higher gene flow (*m*), or lower differentiation than expected given the geographic distances) was observed connecting the eastern localities at Devil's Backbone (28) and Ott's Spring (29) with the Comal Springs complex localities (sites 30–33) to the south, Guadalupe River State Park (23), Honey Creek (24) and Preserve Cave (25) in the northern portion of sampling, and to south westerly localities including Camp Bullis (21, 22). Another corridor connects the western localities of Fessenden Springs (2), Hill Country SNA (3) and Western Kerr (4) with southwestern sites Osborn Springs (5), Albert and Bessie Kronkowsky SNA (8) and Government Canyon (11). Another smaller corridor connects Osborn Springs (5) with Maverick Ranch (14) and Cascade Caverns (15). These areas of higher inferred gene flow roughly parallel the inferred migration paths from treemix (Figure 13) and correspond with the patterns of admixture (Figure 4). There are also several areas that appear as barriers (or areas with lower‐than‐expected gene flow or higher differentiation than expected given the geographic distances). One major barrier appears between Possum Creek Spring (6), Brown Ranch (7) and Salamander Spring (9) on the east side of the barrier and western localities at Fessenden Springs (2), Hill Country SNA (3) and Western Kerr (4). Another barrier separates three proximal localities in the center of our sampling (Guadalupe River State Park (23), Honey Creek (24) and Preserve Cave (25)) from more westerly localities (Possum Creek Spring (6), Brown Ranch (7) and Salamander Spring (9)). In general, the estimated effective migration surface suggests that there are clear deviations from a simple IDB pattern across the sample range of this study which supports the hypothesis that there has been some gene flow during divergence or since populations and lineages have diverged.

## 4 Discussion

The history of salamanders of the *E. neotenes* complex ( *E. neotenes* , *E. latitans*, and *E. pterophila* ) appears to be one of recent diversification among three lineages whose boundaries are obscured by substantial admixture not only among localities we sampled within the species complex, but also with adjacent lineages including *E. nana* , *E. troglodytes*, and *E*. sp. 2 (sensu Devitt et al. (2019)) (Figures 4 and 5). These patterns are consistent with a history of recent divergence accompanied by gene flow.

This conclusion is supported by several lines of evidence: First, differentiation among localities and taxa (measured as *F*_ST_) is low (Figures 2 and 9, Tables S2–S5, Figure 6). Second, clustering analyses reveal prevalent admixture among localities and between nominal species (Figure 4). Third, nominal taxonomy explained only slightly more genetic variation than did geography in an RDA, but overall variance explained was low (Figure 8). Moreover, both taxonomy and geography were important predictors in Bayesian mixed models, but geography alone had greater support than taxonomy alone (Table 2). Fourth, analyses of admixture events revealed evidence of extensive gene exchange both within and among lineages (Figures 5 and 13). These patterns of gene exchange suggest the *E. neotenes* salamanders are, or have been, capable of movement within at least some portions of the Edwards Plateau aquifers. However, while the addition of migration events in the treemix analyses improved our ability to explain variation in allele frequencies, that improvement was small (Figure 13) and it is important to note that the drift tree alone (*m* = 0) explained considerable variance. This indicates that some of the observed admixture might be derived from the recency of divergence and the retention of ancestral polymorphism. Fifth, negative *f*_3_ statistics for all sampling localities supports the inference of gene exchange. Sixth, examination of the estimated effective migration surface shows departures from a simple isolation‐by‐distance pattern which might be expected under a primary divergence scenario. Collectively, these lines of evidence support the hypothesis that the population genetic variation of the *E. neotenes* complex salamanders has been shaped by recent divergence, retention of ancestral polymorphism, and a history of gene exchange.

Both the PCA and clustering analysis provide important insights into the organization of genetic variation within and among localities and lineages. While three loosely organized clusters are apparent within the *E. neotenes* complex (Figures 2 and 4), genomic differentiation among localities and nominal taxa is low (Tables S2–S5, Figure 6) and many localities exhibit admixture (Figures 4 and 5). Importantly, admixture is evident at the type locality for *E. latitans* (Smith and Potter 1946), Cascade Caverns (15). Many localities exhibit admixture from multiple ancestral lineages, with Preserve Cave (25) showing evidence of admixture from five clusters (though the contribution from the *E. nana* cluster is minor). Specific tests of gene exchange using the treemix algorithm, and calculation of *f*_3_ statistics, indicate gene flow and patterns of allele frequency variation among localities that are inconsistent with bifurcating relationships. Thus, a major finding of our research is that the three nominal taxa that have historically been recognized are not clearly differentiated. The lack of morphological characters available to differentiate among these species (Chippindale et al. 2000; Sweet 1984), combined with evidence of extensive admixture among populations and lineages, indicate that the nominal species *E. neotenes* , *E. latitans* , and *E. pterophila* do not represent three independent lineages, but rather form three closely related management units of a single lineage with a history of gene exchange among them.

Despite the evidence of admixture among *E. neotenes* localities, levels of genetic diversity (*θ* and *π*, Figure 7) were marginally lower across the complex compared to populations to the east ( *E. nana* ) and west ( *E. troglodytes* and *E. sp. 2*) and two to three times lower than observed previously in *E. chisholmensis* (Nice et al. 2021). These low levels suggest that contemporary effective population sizes might be comparatively low in *E. neotenes* complex populations.

The inferred patterns of gene exchange provide clues to how *Eurycea* salamanders are, or have been, capable of moving through the aquifers such that the aquifers serve as a conduit for gene exchange. The apparent admixture is unexpected from the perspective that these salamanders are specialized stygobionts and limited to narrow ranges of water temperature and quality (Barr and Babbitt 2002; Bowles, Sanders, and Hansen 2006; Crow et al. 2016). We might have predicted greater isolation and more differentiation based on our understanding of salamander life histories. The evidence of extensive admixture suggests that the aquatic environment in this aquifer system includes, or has included in the recent past, large areas of relatively homogeneous environmental conditions that permit salamander movements and thus gene flow. Additionally, this suggests that there are passages within the aquifer system that allow such movements.

It is important to note the caveat that the analyses presented here cannot discriminate between gene flow that might have occurred during the divergence of the three admixed lineages that we observe in the *E. neotenes* complex and post‐divergence gene flow. More detailed, explicitly demographic models are probably required for this, but might be difficult to apply given the scale of the sampling and the low levels of differentiation among sampled localities. Consequently, the details of this gene exchange and salamander movements remain obscure. What is evident by mapping the inferred migration events (Figure 5) is that gene flow was likely dynamic. Some of the migration events inferred from treemix analysis occurred along the path of known movement of water in the aquifer system in the central Edwards Plateau, which is thought to be generally and primarily from northwest to southeast within and across the constituent aquifers (Maclay 1995). For example, inferred migration from Fesseneden Springs (2) to Western Kerr (4) and from Western Kerr (4) to Cascade Cavern (15) parallels known aquifer flows. However, other migration events do not align with the direction of water flow. For example, inferred migrations from *E. nana* (36, 37, 38) to the *E. neotenes* complex, particularly the southwestern localities (11, 17, 19, 21, 22), is nearly in the opposite direction, as is inferred migration from southwestern *E. neotenes* (11, 17, 19) to *E. troglodytes* (3), and from southwestern *E. neotenes* (17, 19) to Osborn Springs (5). These events suggest that salamander movements via the aquifers might have been dynamic over their history. Major fluctuations in aquifer water levels or unrecognized flow path changes might explain some of these patterns. Alternatively, the patterns we have elucidated might suggest that *Eurycea* movements are not constrained by aquifer water flow paths. However, it is also important to note that pathways for salamanders in the aquifers are undoubtedly quite complex and arrows plotted on a surface map (Figure 5) most assuredly represent an incomplete picture.

Similarly, inferred migration events from treemix also provide an incomplete picture of the history of gene exchange in these salamanders. Eight migration events inferred from treemix analysis explained a small proportion of the variance in allele frequencies among populations (Figure 12) but likely does not represent the full picture of gene flow. There were likely many more events, perhaps of smaller magnitude in terms of numbers of migrants or distance of migrations, that were not detected. The migration events inferred from treemix do not fully comport with the more extensive patterns of admixture observed in clustering analysis (Figure 4), which suggests that even more migrations have occurred in the history of these populations or that some of the admixture occurred during divergence. In addition, replicate runs for each model produced a range of different solutions (Figure 12) perhaps suggesting that many small migration events are also compatible with the data. Indeed, *f*_3_ statistics indicate that gene flow was detectable in all localities, emphasizing again that strictly bifurcating relationships are insufficient to capture the history of divergence at all localities.

The results reported here greatly expand what was known from previous work. Chippindale et al. (2000) used 22 allozyme loci and one mitochondrial DNA (mtDNA) locus to survey genetic variation across central Texas *Eurycea* and documented especially low levels of differentiation among the *E. neotenes* complex taxa and localities, similar to results reported here. However, Chippindale et al. (2000) deemed the observed differentiation to be of sufficient magnitude to support delineating the nominal species within the *E. neotenes* complex ( *E. neotenes* , *E. pterophila* , *E. latitans* , and *E. tridentifera* ). Using mtDNA and a single‐copy nuclear marker, Lucas et al. (2009) found modest amounts of sequence divergence among six localities from the eastern portion of the *E. neotenes* complex, though levels of differentiation estimated as *ϕ*_ST_ (Excoffier, Smouse, and Quattro 1992) indicated more substantial allele frequency variation than reported here (pairwise *ϕ*_ST_ ranged from 0.249 to 0.924 (Lucas et al. 2009)). These differences might reflect the limitations of a small number of sites or loci and the types of markers used by the previous studies. Bendik et al. (2013) used mtDNA for a phylogenetic exploration of *Eurycea* south of the Colorado River, including the *E. neotenes* complex. They concluded that while there was clear structure, there was also evidence of admixed populations (especially compared to morphology) resulting from incomplete lineage sorting or gene exchange.

Our results comprise the most detailed and geographically intensive investigation of potential gene flow in the stygobiont salamanders of central Texas. We provide evidence that the aquifer might contain important pathways for gene flow among localities and facilitate connectivity. While it is currently difficult to distinguish historical and contemporary gene flow, the picture for the *E. neotenes* complex is one of widespread gene flow and possibly a complicated history of fluctuating gene flow. From a conservation perspective, these results indicate that water flow throughout the aquifer, not just at springs, might be important for the persistence of salamander populations and the maintenance of genetic variation throughout the species complex. Underground conduits allow genetic variation to be exchanged and could play an important future role in the ability of these salamanders to respond to increasing human impacts. The level of admixture among seemingly isolated populations of salamanders observed at springs and in caves is surprising but highlights the potential connectivity of populations of the larger community of stygobionts, including several other threatened or endangered taxa, over relatively large areas and over time. Comparative investigations of gene exchange will be needed to determine whether and how other stygobionts utilize the aquifer connections and whether life history variation predicts levels of connectivity. Our results also suggest that conserving the underground connections between populations of stygobionts might be important for ensuring the persistence of these aquatic populations in the face of increasing human impacts.

## Author Contributions

**Chris C. Nice:** conceptualization (equal), data curation (lead), formal analysis (lead), funding acquisition (equal), writing – original draft (lead), writing – review and editing (equal). **Katherine L. Bell:** conceptualization (supporting), formal analysis (supporting), writing – review and editing (equal). **Zachariah Gompert:** conceptualization (supporting), formal analysis (supporting), writing – review and editing (equal). **Lauren K. Lucas:** conceptualization (supporting), data curation (supporting), formal analysis (supporting), writing – review and editing (equal). **James R. Ott:** conceptualization (supporting), data curation (supporting), writing – review and editing (equal). **Ruben U. Tovar:** data curation (supporting), writing – review and editing (equal). **Paul Crump:** conceptualization (equal), data curation (supporting), funding acquisition (equal), writing – review and editing (equal). **Peter H. Diaz:** conceptualization (equal), data curation (equal), funding acquisition (equal), writing – review and editing (equal).

## Conflicts of Interest

The authors declare no conflicts of interest.

## Supporting information


Data S1.

## Data Availability

The DNA sequence data analyzed in this manuscript have been archived on NCBI's SRA (PRJNA1057889). Genotype estimates and a predictors file for Redundancy Analysis and BLMM have been archived on Dryad (https://doi.org/10.5061/dryad.3r2280gpx).
