# Supplementary material for: Extensive Admixture Among Karst‐Obligate Salamanders Reveals Evidence of Recent Divergence and Gene Exchange Through Aquifers
Source: Ecol Evol. 2025 Jan 9;15(1):e70785. doi: 10.1002/ece3.70785 (PMC11717725; doi:10.1002/ece3.70785)
Supplement: Supplementary file 1 — Data S1. [file ECE3-15-e70785-s001.pdf]

Supplemental Material

Extensive admixture among karst-obligate salamanders reveals evidence of recent divergence and gene exchange through aquifers

Chris C. Nice[1], Katherine L. Bell[2], Zachariah Gompert[3], Lauren K. Lucas[3], James R. Ott [1], Ruben U. Tovar[4], Paul Crump[5] and Pete Diaz[6]

[1] Department of Biology, Population and Conservation Biology Program, Texas State University, San Marcos, Texas 78666, USA
[2] Department of Biology, University of Nevada, Reno, NV 89557, USA [3] Department of Biology, Utah State University, Logan, UT 84322, USA
[4] Department of Integrative Biology, The University of Texas at Austin, Texas 78712, USA
[5] Nongame and Rare Species Program, Wildlife Division Texas Parks and Wildlife Department, Austin, Texas 78744, USA
[6] United States Fish and Wildlife Service, Texas Fish and Wildlife Conservation Office, San Marcos, Texas 78666, USA

Corresponding author: Chris Nice
Department of Biology
Texas State University
San Marcos, TX 78666, USA
ccnice@txstate.edu

Keywords: *genomic differentiation, gene flow, population genomics,* Eurycea*, Plethodontidae*

Running title: *Population Genetics of* Eurycea *salamanders*

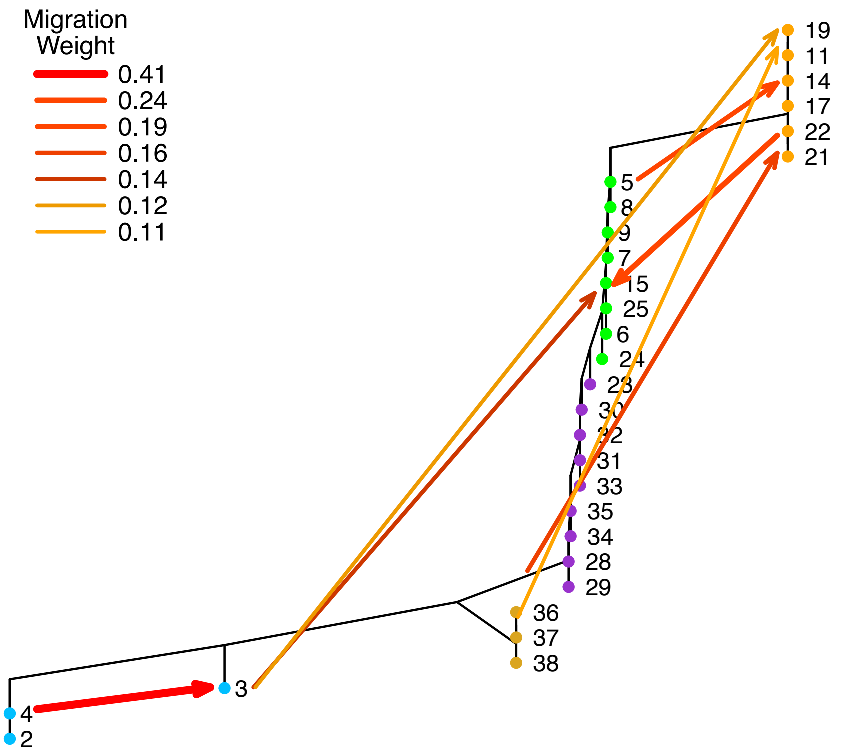

Supplementary Figure 1: Treemix tree of salamander localities with seven migration events (m = 7). Locality numbers follow Table 1. Locality 2 serves as the outgroup.

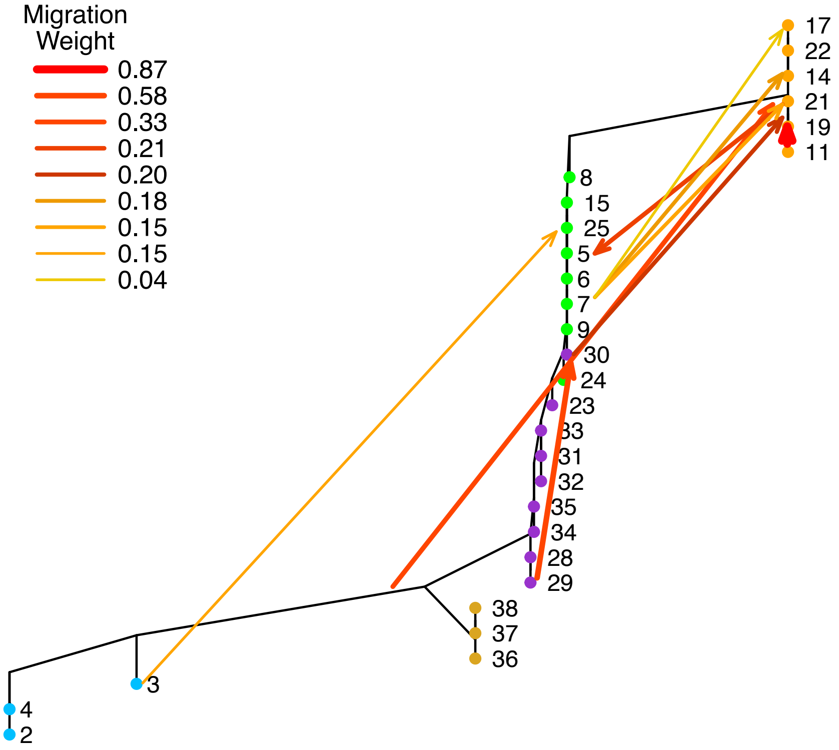

Supplementary Figure 2: Treemix tree of salamander localities with nine migration events (m = 9). Locality numbers follow Table 1. Locality 2 serves as the outgroup.

Supplementary Table 1: Detailed locality information.

site	locality	n	lat	long	nominal species	year sampled
1	Nueces 335	2	29.8129	-100.0173	troglodytes	2021
2	Stockman/Fessenden	33	30.16653	-99.34269	sp. 2	2005
3	Hill Country SNA	11	29.628667	-99.177944	troglodytes	2020
4	Western Kerr	14	30.1	-99	sp. 2	2021
5	Osborn Spring	13	29.684866	-98.877808	neotenes	2020
6	Possum Creek Spring	14	29.878388	-98.869046	neotenes	2020
7	Brown Ranch	42	29.83434	-98.86147	neotenes	2020
8	Albert and Bessie Kronkosky	44	29.768174	-98.860135	neotenes	2020
9	Salamander Spring	16	29.842002	-98.858971	neotenes	2021
10	Pecan Springs Cave	1	29.634165	-98.852694	neotenes	1993
11	Government Canyon	22	29.584722	-98.761111	neotenes	2020
12	Mueller's Ranch	2	29.706848	-98.753168	neotenes	1990
13	Helotes Creek Spring	3	29.6375	-98.694444	neotenes	1990
14	Maverick Ranch	28	29.707911	-98.694008	neotenes	2021
15	Cascade Caverns	28	29.763788	-98.678924	latitans	2020
16	Pfeiffer Ranch	4	29.762222	-98.666389	latitans	1992
17	Leahs Spring	9	29.637204	-98.665931	neotenes	2021
18	Peavey's Spring	1	30.091667	-98.658333	latitans	1990
19	Leon Springs	29	29.662974	-98.637397	neotenes	2020
20	Badweather Pit	3	29.755833	-98.620278	latitans	1990
21	Camp Bullis Stealth	8	29.660654	-98.599256	neotenes	2021
22	Camp Bullis Sharon	12	29.66247	-98.595703	neotenes	2021
23	Guadalupe River State Park	22	29.87161	-98.49318	latitans	2020
24	Honey Creek SNA Springs	32	29.858401	-98.485567	latitans	2019/2020
25	Preserve Cave	10	29.864894	-98.477703	latitans	2020
26	Sattler's Deep Pit	2	29.9282	-98.45104	latitans	1994
27	Rebecca Spring	2	29.924194	-98.374472	latitans	2021
28	Devil's Backbone	18	29.925278	-98.155	pterophila	2005
29	Ott's spring	17	29.922778	-98.150833	pterophila	2005
30	Hueco Springs	15	29.765556	-98.141944	pterophila	2005
31	Comal Spr Run One	14	29.712458	-98.137464	pterophila	2005
32	Comal Spr Run Three	19	29.7138	-98.136369	pterophila	2005
33	Comal SPr Spring Island	22	29.717475	-98.132228	pterophila	2005
34	Jacobs Well	21	30.034167	-98.122222	pterophila	2005
35	Fern Bank	35	29.993889	-97.995833	pterophila	2005
36	San Marcos below dam	18	29.89	-97.933611	nana	2005
37	San Marcos Diversion	16	29.893186	-97.931878	nana	2005
38	San Marcos Hotel	15	29.893889	-97.93	nana	2005

Supplementary Table 2: Pairwise F_{ST} values among all sites with sample sizes of at least 8 individuals (below diagonal) and 95% permutational confidence intervals (above diagonal). Site numbering follows Table 1 and Figure 1

**site:**	**2**	**3**	**4**	**5**	**6**	**7**	**8**	**9**
**2**	0	0.031-0.034	0.010-0.011	0.114-0.123	0.117-0.126	0.114-0.122	0.112-0.121	0.116-0.126
**3**	0.032	0	0.033-0.036	0.081-0.088	0.085-0.092	0.081-0.088	0.080-0.087	0.085-0.091
**4**	0.011	0.034	0	0.116-0.125	0.119-0.128	0.115-0.124	0.113-0.122	0.118-0.127
**5**	0.118	0.085	0.12	0	0.011-0.012	0.009-0.010	0.008-0.008	0.012-0.013
**6**	0.122	0.089	0.124	0.012	0	0.006-0.007	0.007-0.008	0.009-0.009
**7**	0.118	0.085	0.12	0.009	0.006	0	0.004-0.004	0.005-0.006
**8**	0.116	0.083	0.118	0.008	0.008	0.004	0	0.006-0.007
**9**	0.121	0.088	0.123	0.012	0.009	0.005	0.007	0
**11**	0.121	0.085	0.123	0.027	0.034	0.031	0.028	0.034
**14**	0.115	0.08	0.117	0.019	0.025	0.02	0.018	0.022
**15**	0.112	0.078	0.113	0.01	0.01	0.007	0.007	0.01
**17**	0.125	0.088	0.126	0.03	0.037	0.033	0.03	0.035
**19**	0.118	0.083	0.12	0.025	0.032	0.028	0.025	0.03
**21**	0.122	0.086	0.124	0.026	0.031	0.027	0.025	0.029
**22**	0.119	0.083	0.121	0.024	0.03	0.025	0.023	0.027
**23**	0.109	0.078	0.111	0.012	0.011	0.009	0.009	0.011
**24**	0.112	0.079	0.113	0.011	0.009	0.007	0.007	0.009
**25**	0.099	0.067	0.101	0.02	0.022	0.019	0.018	0.022
**28**	0.114	0.082	0.116	0.023	0.022	0.02	0.02	0.023
**29**	0.114	0.082	0.116	0.022	0.022	0.02	0.019	0.023
**30**	0.11	0.078	0.112	0.014	0.013	0.011	0.011	0.014
**31**	0.113	0.082	0.115	0.018	0.017	0.015	0.015	0.018
**32**	0.111	0.08	0.113	0.017	0.016	0.013	0.013	0.016
**33**	0.112	0.08	0.113	0.016	0.016	0.013	0.013	0.016
**34**	0.114	0.082	0.115	0.02	0.019	0.017	0.017	0.02
**35**	0.11	0.078	0.112	0.017	0.016	0.014	0.014	0.017
**36**	0.107	0.079	0.11	0.049	0.05	0.047	0.046	0.049
**37**	0.108	0.08	0.11	0.049	0.051	0.047	0.046	0.05
**38**	0.108	0.08	0.11	0.049	0.05	0.047	0.046	0.049

Supplementary Table 3: Pairwise F_{ST} values (continued) from Supplementary Table 2. Pairwise F_{ST} values among all sites with sample sizes of at least 8 individuals (below diagonal) and 95% permutational confidence intervals (above diagonal). Site numbering follows Table 1 and Figure 1

**site:**	**11**	**14**	**15**	**17**	**19**	**21**	**22**	**23**
**2**	0.116-0.125	0.111-0.120	0.108-0.116	0.120-0.129	0.114-0.123	0.118-0.126	0.115-0.123	0.105-0.113
**3**	0.081-0.088	0.077-0.083	0.075-0.081	0.085-0.092	0.079-0.086	0.083-0.089	0.080-0.086	0.075-0.081
**4**	0.118-0.127	0.113-0.121	0.109-0.117	0.122-0.131	0.116-0.124	0.120-0.128	0.116-0.125	0.107-0.115
**5**	0.026-0.028	0.018-0.020	0.010-0.010	0.029-0.032	0.023-0.026	0.025-0.027	0.023-0.025	0.012-0.013
**6**	0.032-0.036	0.024-0.026	0.010-0.011	0.036-0.039	0.030-0.033	0.030-0.032	0.028-0.031	0.010-0.011
**7**	0.029-0.033	0.019-0.021	0.007-0.007	0.031-0.034	0.026-0.030	0.026-0.028	0.024-0.026	0.008-0.009
**8**	0.027-0.030	0.017-0.019	0.006-0.007	0.029-0.032	0.024-0.027	0.024-0.026	0.022-0.024	0.008-0.009
**9**	0.032-0.036	0.021-0.023	0.009-0.010	0.033-0.036	0.028-0.032	0.028-0.030	0.026-0.028	0.011-0.012
**11**	0	0.008-0.008	0.021-0.023	0.010-0.011	0.005-0.006	0.013-0.014	0.010-0.011	0.029-0.032
**14**	0.008	0	0.013-0.015	0.010-0.010	0.006-0.006	0.011-0.011	0.008-0.008	0.021-0.023
**15**	0.022	0.014	0	0.023-0.025	0.018-0.020	0.019-0.021	0.017-0.019	0.010-0.011
**17**	0.01	0.01	0.024	0	0.008-0.009	0.015-0.016	0.012-0.013	0.032-0.035
**19**	0.006	0.006	0.019	0.009	0	0.011-0.012	0.008-0.008	0.027-0.029
**21**	0.013	0.011	0.02	0.016	0.011	0	0.011-0.012	0.027-0.029
**22**	0.01	0.008	0.018	0.012	0.008	0.012	0	0.025-0.028
**23**	0.031	0.022	0.01	0.033	0.028	0.028	0.026	0
**24**	0.028	0.019	0.008	0.03	0.025	0.026	0.024	0.007
**25**	0.03	0.023	0.015	0.033	0.027	0.028	0.026	0.018
**28**	0.037	0.03	0.02	0.04	0.035	0.036	0.034	0.016
**29**	0.037	0.03	0.02	0.04	0.034	0.036	0.034	0.015
**30**	0.031	0.023	0.012	0.034	0.028	0.029	0.027	0.009
**31**	0.036	0.028	0.017	0.039	0.034	0.035	0.032	0.012
**32**	0.034	0.026	0.015	0.037	0.031	0.032	0.03	0.011
**33**	0.034	0.026	0.015	0.037	0.031	0.032	0.03	0.011
**34**	0.037	0.028	0.018	0.039	0.034	0.035	0.033	0.013
**35**	0.033	0.025	0.015	0.036	0.031	0.031	0.029	0.01
**36**	0.056	0.048	0.044	0.058	0.053	0.055	0.052	0.042
**37**	0.056	0.049	0.045	0.058	0.053	0.056	0.053	0.042
**38**	0.056	0.048	0.044	0.058	0.053	0.055	0.052	0.042

Supplementary Table 4: Pairwise F_{ST} values (continued) from Supplementary Table 3. Pairwise F_{ST} values among all sites with sample sizes of at least 8 individuals (below diagonal) and 95% permutational confidence intervals (above diagonal). Site numbering follows Table 1 and Figure 1

**sites:**	**24**	**25**	**28**	**29**	**30**	**31**	**32**	**33**
**2**	0.107-0.116	0.095-0.103	0.110-0.118	0.109-0.118	0.106-0.114	0.109-0.117	0.107-0.115	0.107-0.116
**3**	0.076-0.082	0.065-0.070	0.079-0.085	0.079-0.085	0.075-0.081	0.079-0.085	0.077-0.083	0.077-0.083
**4**	0.109-0.117	0.097-0.105	0.112-0.120	0.111-0.120	0.107-0.116	0.111-0.120	0.109-0.117	0.109-0.118
**5**	0.010-0.011	0.020-0.021	0.022-0.024	0.021-0.023	0.013-0.014	0.018-0.019	0.016-0.017	0.016-0.017
**6**	0.009-0.010	0.021-0.022	0.021-0.023	0.021-0.022	0.012-0.013	0.017-0.018	0.015-0.016	0.015-0.016
**7**	0.006-0.007	0.018-0.020	0.019-0.021	0.019-0.021	0.010-0.011	0.015-0.016	0.013-0.014	0.013-0.014
**8**	0.006-0.007	0.018-0.019	0.019-0.021	0.019-0.020	0.010-0.011	0.015-0.016	0.013-0.014	0.013-0.014
**9**	0.009-0.010	0.021-0.022	0.022-0.024	0.022-0.024	0.013-0.014	0.018-0.019	0.016-0.017	0.016-0.017
**11**	0.027-0.030	0.028-0.031	0.036-0.039	0.035-0.039	0.029-0.033	0.034-0.038	0.033-0.036	0.032-0.036
**14**	0.018-0.020	0.022-0.024	0.028-0.031	0.028-0.031	0.022-0.024	0.027-0.029	0.024-0.027	0.024-0.027
**15**	0.008-0.008	0.014-0.015	0.019-0.021	0.019-0.021	0.011-0.012	0.016-0.017	0.014-0.015	0.014-0.015
**17**	0.029-0.032	0.031-0.034	0.038-0.042	0.039-0.042	0.033-0.035	0.037-0.041	0.035-0.039	0.035-0.039
**19**	0.024-0.027	0.026-0.028	0.033-0.036	0.033-0.036	0.027-0.030	0.032-0.035	0.030-0.033	0.030-0.033
**21**	0.024-0.027	0.027-0.029	0.035-0.038	0.035-0.037	0.028-0.030	0.033-0.036	0.031-0.034	0.031-0.034
**22**	0.022-0.025	0.025-0.027	0.033-0.035	0.032-0.035	0.026-0.028	0.031-0.034	0.029-0.031	0.029-0.032
**23**	0.007-0.007	0.017-0.018	0.015-0.017	0.015-0.016	0.009-0.009	0.012-0.013	0.010-0.011	0.010-0.011
**24**	0	0.014-0.015	0.017-0.018	0.016-0.018	0.009-0.009	0.013-0.014	0.011-0.012	0.011-0.012
**25**	0.015	0	0.025-0.027	0.024-0.026	0.017-0.019	0.022-0.024	0.020-0.022	0.020-0.022
**28**	0.017	0.026	0	0.006-0.007	0.012-0.013	0.015-0.017	0.015-0.016	0.015-0.016
**29**	0.017	0.025	0.007	0	0.012-0.013	0.015-0.016	0.014-0.015	0.014-0.016
**30**	0.009	0.018	0.013	0.012	0	0.011-0.012	0.010-0.010	0.010-0.010
**31**	0.013	0.023	0.016	0.015	0.011	0	0.006-0.007	0.007-0.007
**32**	0.012	0.021	0.015	0.015	0.01	0.006	0	0.006-0.006
**33**	0.011	0.021	0.015	0.015	0.01	0.007	0.006	0
**34**	0.014	0.024	0.018	0.017	0.013	0.016	0.015	0.015
**35**	0.012	0.021	0.015	0.014	0.01	0.013	0.012	0.012
**36**	0.043	0.045	0.045	0.044	0.041	0.045	0.044	0.044
**37**	0.043	0.045	0.045	0.045	0.042	0.045	0.044	0.044
**38**	0.043	0.045	0.045	0.045	0.042	0.045	0.044	0.044

Supplementary Table 5: Pairwise F_{ST} values (continued) from Supplementary Table 4. Pairwise F_{ST} values among all sites with sample sizes of at least 8 individuals (below diagonal) and 95% permutational confidence intervals (above diagonal). Site numbering follows Table 1 and Figure 1

**sites:**	**34**	**35**	**36**	**37**	**38**
	34	35	36	37	38
**2**	0.109-0.118	0.105-0.114	0.103-0.111	0.104-0.112	0.103-0.112
**3**	0.079-0.085	0.075-0.081	0.076-0.082	0.077-0.083	0.077-0.083
**4**	0.111-0.120	0.108-0.116	0.106-0.114	0.106-0.115	0.106-0.115
**5**	0.019-0.021	0.016-0.018	0.047-0.051	0.047-0.051	0.047-0.051
**6**	0.018-0.020	0.016-0.017	0.048-0.053	0.049-0.053	0.048-0.052
**7**	0.016-0.018	0.013-0.015	0.045-0.049	0.045-0.049	0.045-0.049
**8**	0.016-0.018	0.013-0.014	0.044-0.048	0.045-0.049	0.044-0.048
**9**	0.019-0.021	0.016-0.017	0.047-0.052	0.047-0.052	0.047-0.051
**11**	0.035-0.038	0.032-0.035	0.053-0.058	0.053-0.058	0.053-0.058
**14**	0.027-0.030	0.024-0.026	0.046-0.050	0.047-0.051	0.046-0.050
**15**	0.017-0.019	0.014-0.016	0.043-0.046	0.043-0.047	0.042-0.046
**17**	0.038-0.041	0.034-0.037	0.055-0.060	0.055-0.060	0.055-0.060
**19**	0.032-0.035	0.029-0.032	0.051-0.055	0.051-0.055	0.050-0.055
**21**	0.033-0.036	0.030-0.033	0.053-0.057	0.053-0.058	0.053-0.057
**22**	0.031-0.034	0.028-0.030	0.050-0.055	0.050-0.055	0.050-0.055
**23**	0.012-0.013	0.010-0.011	0.040-0.044	0.041-0.044	0.040-0.044
**24**	0.014-0.015	0.011-0.012	0.041-0.045	0.041-0.045	0.041-0.045
**25**	0.023-0.025	0.020-0.022	0.043-0.047	0.044-0.047	0.043-0.047
**28**	0.017-0.019	0.015-0.016	0.043-0.047	0.044-0.047	0.043-0.047
**29**	0.016-0.018	0.014-0.015	0.043-0.046	0.043-0.047	0.043-0.047
**30**	0.012-0.013	0.010-0.011	0.040-0.043	0.040-0.044	0.040-0.043
**31**	0.015-0.017	0.013-0.014	0.043-0.047	0.043-0.047	0.043-0.047
**32**	0.014-0.016	0.012-0.013	0.042-0.045	0.042-0.046	0.042-0.046
**33**	0.014-0.016	0.012-0.013	0.042-0.046	0.042-0.046	0.042-0.046
**34**	0	0.005-0.005	0.040-0.044	0.040-0.044	0.041-0.044
**35**	0.005	0	0.037-0.040	0.037-0.041	0.037-0.041
**36**	0.042	0.039	0	0.005-0.006	0.006-0.006
**37**	0.042	0.039	0.005	0	0.006-0.006
**38**	0.042	0.039	0.006	0.006	0

Supplementary Table 6: Results of three-population test of admixture. These are abbreviated results with only the most negative f_3 statistic for each locality as the target of admixture presented along with the source localities for that specific test. For localities 31-33 and 36-38, localities within large spring complexes (Comal Springs and San Marcos Springs, respectively), the most negative f_3 statistics were observed with source localities located in the same spring complex. Below the line we report the second-most negative f_3 statistics for these sites (i.e. with one source population from outside the local spring complex), and the most negative f_3 statistic in which both source populations are outside of the local spring complex. Tests in bold are significant at an arbitrary $p < 0.000001$. Only localities with $n \geq 8$ were included in these tests.

**Target**	**Source 1**	**Source 2**	**f_3**	**Std. Error**	**Z-score**
**2**	**3**	**4**	**-0.00229295**	**0.000106912**	**-21.4471**
**3**	**2**	**19**	**-0.00957279**	**0.000277759**	**-34.4644**
**4**	**2**	**3**	**-0.00353063**	**0.000114193**	**-30.918**
**5**	**8**	**15**	**-0.00636996**	**7.62176e-05**	**-83.576**
**6**	**7**	**24**	**-0.00638172**	**5.60728e-05**	**-113.811**
**7**	**8**	**9**	**-0.00236467**	**2.95913e-05**	**-79.9111**
**8**	**5**	**7**	**-0.00225418**	**4.02737e-05**	**-55.9714**
**9**	**7**	**8**	**-0.0055665**	**4.44625e-05**	**-125.195**
**11**	**14**	**19**	**-0.00355891**	**5.39144e-05**	**-66.0104**
**14**	**15**	**19**	**-0.00452096**	**6.79268e-05**	**-66.5564**
**15**	**8**	**25**	**-0.0040262**	**7.09935e-05**	**-56.7122**
**17**	**14**	**19**	**-0.00919705**	**7.65707e-05**	**-120.112**
**19**	**11**	**14**	**-0.00349781**	**3.71554e-05**	**-94.1402**
**21**	**14**	**22**	**-0.010668**	**9.59334e-05**	**-111.202**
**22**	**14**	**21**	**-0.00868404**	**7.10733e-05**	**-122.184**
**23**	**24**	**30**	**-0.00410975**	**5.4395e-05**	**-75.5538**
**24**	**15**	**23**	**-0.00286774**	**5.34846e-05**	**-53.6182**
**25**	**3**	**15**	**-0.0106783**	**0.000196798**	**-54.2601**
**28**	**29**	**30**	**-0.00506417**	**6.39127e-05**	**-79.2358**
**29**	**28**	**30**	**-0.00563487**	**5.82594e-05**	**-96.7203**
**30**	**24**	**35**	**-0.00610045**	**6.66204e-05**	**-91.5703**
**31**	**32**	**33**	**-0.00663148**	**5.03875e-05**	**-131.61**
**32**	**31**	**33**	**-0.00536484**	**4.2545e-05**	**-126.098**
**33**	**31**	**32**	**-0.00411446**	**4.88667e-05**	**-84.1977**
**34**	**23**	**35**	**-0.00368665**	**7.33839e-05**	**-50.2379**
**35**	**23**	**34**	**-0.00304483**	**5.48816e-05**	**-55.48**
**36**	**37**	**38**	**-0.00484558**	**3.77361e-05**	**-128.407**
**37**	**36**	**38**	**-0.00545892**	**3.82996e-05**	**-142.532**
**38**	**36**	**37**	**-0.00573825**	**4.21412e-05**	**-136.167**
**31**	**32**	**35**	**-0.00659928**	**6.00073e-05**	**-109.975**
**31**	**23**	**29**	**-0.00538465**	**1.23327e-04**	**-43.6617**
**32**	**24**	**31**	**-0.00563032**	**5.22206e-05**	**-107.818**
**32**	**7**	**29**	**-0.00446188**	**1.23057e-04**	**-36.25880**
**33**	**30**	**32**	**-0.00431445**	**5.20231e-05**	**-82.9335**
**33**	**7**	**29**	**-0.003547700**	**1.29704e-04**	**-27.35230**
**36**	**35**	**38**	**-0.00490134**	**6.40483e-05**	**-76.5256**
36	34	6	0.00232263	4.79394e-04	4.84493
**37**	**35**	**36**	**-0.00529426**	**6.64943e-05**	**-79.61980**
37	2	9	0.00211488	4.70187e-04	4.49795
**38**	**35**	**36**	**-0.00568249**	**6.85136e-05**	**-82.93960**
38	2	9	0.00169419	4.70917e-04	3.59764
